# Synthesis, Characterization, Antioxidant Evaluation, Cytotoxicity Studies, and Molecular Docking of Novel Sulfonamide Derivatives

**DOI:** 10.1002/jbt.70981

**Published:** 2026-06-23

**Authors:** Ouissal Bouraoui, Güldeniz Şekerci, Khaled Mesbah, James A. Ezugwu, Rachid Benkiniouar, Suat Tekin, Fatümetüzzehra Küçükbay, Houssem Boulebd, Hasan Küçükbay

**Affiliations:** ^1^ Laboratory of Natural Products of Plant Origin and Organic Synthesis Frères Mentouri University Constantine 1 Constantine Algeria; ^2^ Department of Chemistry, Faculty of Arts and Sciences İnonu University Malatya Turkey; ^3^ Department of Physiology, Faculty of Medicine İnönü University Malatya Turkey; ^4^ Department of Pure and Industrial Chemistry University of Nigeria Nsukka Nigeria; ^5^ Department of Basic Pharmaceutical Sciences, İnonu University Faculty of Pharmacy Malatya Turkey; ^6^ Laboratory of Synthesis of Molecules with Biological Interest, Department of Chemistry, Faculty of Exact Sciences University Frères Mentouri Constantine 1 Constantine Algeria

**Keywords:** A2780, antioxidant activity, cytotoxicity, DPPH assay, LNCaP, molecular docking, MTT assay, Schiff base, sulfonamide derivatives

## Abstract

A series of novel sulfonamide derivatives (**4a**–**i**) was synthesized via a three‐step sequence involving Schiff base formation, sodium borohydride reduction to the corresponding secondary amines (**2a**–**i**), and subsequent treatment with *p*‐toluenesulfonyl chloride in the presence of sodium carbonate. The structures of all intermediates and final products were unambiguously confirmed by FT‐IR, ^1^H NMR, ^13^C NMR, and HRMS analyses. The antioxidant activity of the target compounds was assessed using the DPPH radical scavenging assay with BHT as a reference standard. Among the synthesized derivatives, compound **4d** exhibited the most pronounced antioxidant activity, surpassing BHT at concentrations of 37.5 and 62.5 µg/mL. The cytotoxicity of the compounds was evaluated against human ovarian carcinoma (A2780) and prostate cancer (LNCaP) cell lines using the MTT assay, with docetaxel as a positive control. Compounds **4g**, **4h**, and **4i** demonstrated the highest cytotoxic potency against both cell lines, with compound **4h** displaying a log IC_50_ value of 1.343 µg/mL against LNCaP cells. Structure–activity relationship analysis suggests that the methoxy substituent enhances cytotoxic activity, likely through increased lipophilicity and electron‐donating effects facilitating interactions with biological targets. Molecular docking studies were performed to further rationalize the observed biological activities.

## Graphical abstract

A series of novel sulfonamide derivatives (**4a**–**i**) were synthesized and characterized. Compound **4d** showed significant antioxidant activity, while compounds **4g**, **4h**, and **4i** exhibited high cytotoxicity against ovarian and prostate cancer cells, particularly **4h** with a log IC_50_ of 1.343 µg/mL. Structure‐activity relationships were explored through molecular docking.

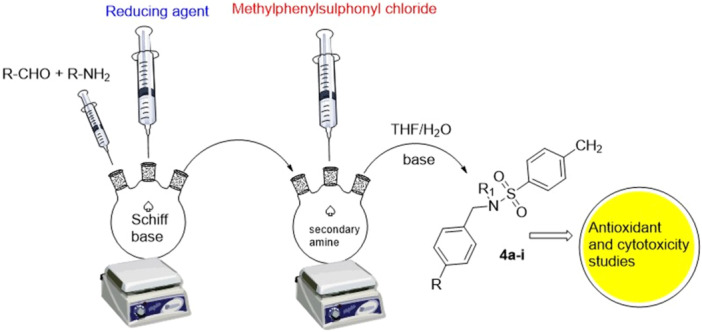

## 1 Introduction

Sulfonamides constitute one of the most versatile and pharmacologically significant classes of organic compounds, with a rich history dating back to the discovery of Prontosil in the 1930s, which heralded the modern era of antibacterial chemotherapy [1]. The sulfonamide moiety has since been recognized as a privileged pharmacophore, appearing in numerous therapeutic agents spanning antibacterial, anticancer, antiviral, anti‐inflammatory, CA enzyme inhibitor and diuretic drug classes [2, 3, 4, 5, 6]. In recent years, considerable attention has been directed toward the design and synthesis of novel sulfonamide derivatives endowed with multifunctional biological properties, particularly antioxidant and anticancer activities, which remain pressing therapeutic needs in contemporary medicinal chemistry [7].

Oxidative stress, arising from an imbalance between reactive oxygen species (ROS) production and the cellular antioxidant defense mechanisms, has been implicated in the pathogenesis of a wide spectrum of chronic diseases including cancer, cardiovascular disorders, neurodegenerative diseases, and diabetes [8]. The development of potent synthetic antioxidants capable of scavenging free radicals represents a viable strategy for mitigating oxidative damage. In parallel, the global burden of cancer continues to escalate, underscoring the urgent need for the discovery of novel cytotoxic agents with improved selectivity and efficacy [4]. We report here in the preparation of a series of novel sulfonamide derivatives (**4a**–**i**) through a sequential Schiff base formation–reduction–sulfonylation strategy. The synthesized compounds were fully characterized by spectroscopic methods and evaluated for their antioxidant activity using the DPPH radical scavenging assay and for their in vitro cytotoxicity against human ovarian carcinoma (A2780) and prostate cancer (LNCaP) cell lines using the MTT assay. Molecular docking studies were additionally performed to provide mechanistic insights into the observed biological activities [9, 10].

## 2 Experimental Section

### 2.1 Chemical Synthesis

#### 2.1.1 General Information

All of the analytical‐grade chemicals and solvents used were acquired from Sigma Aldrich. Using tetramethylsilane as an internal standard, ^1^H NMR and ^13^C NMR spectra were captured in dimethyl sulfoxide (DMSO‐d6)‐d6 using Advance 400 and 101 MHz spectrometers. Thermo Scientific Orbitrap mass spectrometer used to obtain the mass of the compounds. Recorded FTIR spectra were on KBr, (cm^−1^). A Gallenkamp brand melting point determination device was used to obtain the melting point of the compounds. Every experiment was conducted at the Department of Chemistry, Inonu University in Malatya, Turkey.

#### 2.1.2 General Procedure for the Synthesis of Schiff Base

Schiff base intermediates (**1a**–**i**) were synthesized as reported literature [11].

#### 2.1.3 General Procedure for the Preparation of Secondary Amine (2a–i)

To a mixture of Schiff base (**1a**–**i**) in MeOH at 0°C was added NaBH_4_ by portions and allowed to stirred for 5 h. Upon the completion of the reaction, the MeOH was evaporated and crushed ice was added to precipitate the product. The product was filtered washed with cold water to afford (**2a**–**I**) [12].

##### 2.1.3.1 *N*‐(4‐Bromobenzyl)−4‐chloroaniline (**2a**)

Yield: 88%, m.p:. 78.2–78.9°C. ^1^H NMR (400 MHz, CDCl_3_) δ 7.49 (d, *J* = 8.2 Hz, 2H, Ar‐*H*), 7.25 (d, *J* = 8.1 Hz, 2H, Ar‐*H*), 7.13 (d, *J* = 8.6 Hz, 2H, Ar‐*H*), 6.55 (d, *J* = 8.6 Hz,2H, Ar‐*H*), 4.30 (s, 2H, –C *H*_2_NH–). ^13^C NMR (101 MHz, CDCl_3_) δ 146.2, 137.9, 131.8, 129.2, 129.0, 122.5, 121.1, 114.1 (Ar‐*C*), 47.8 (*C* H_2_NH–).

##### 2.1.3.2 *N*‐(4‐Bromobenzyl)−4‐methylaniline (**2b**)

Yield: 88%, m.p:. 64.3–64.8°C. ^1^H NMR (400 MHz, CDCl_3_) δ 7.48 (d, *J* = 8.3 Hz, 2H, Ar‐*H*), 7.27 (d, *J* = 8.2 Hz, 2H, Ar‐*H*), 7.02 (d, *J* = 8.2 Hz, 2H, Ar‐*H*), 6.57 (d, *J* = 8.3 Hz, 2H, Ar‐*H*), 4.30 (br, 2H, –C *H*_2_NH–)), 2.27(s, 3H, C *H*_*3*_Ph–). ^13^C NMR (101 MHz, CDCl_3_) δ 145.4, 138.7, 131.7, 129.8, 129.1, 120.9, 113.1 (Ar‐*C*), 48.1(–*C* H_2_NH), 20.4 (*C* H_3_Ph–).

##### 2.1.3.3 *N*‐(4‐Chlorobenzyl)−4‐chloroaniline (**2c**)

Yield: 81%, m.p:. 72.1–72.8°C. ^1^H NMR (400 MHz, CDCl_3_) δ 7.32 (q, *J* = 8.5 Hz, 4H, Ar‐*H*), 7.14 (d, *J* = 8.7 Hz, 2H, Ar‐*H*), 6.55 (d, *J* = 8.7 Hz, 2H, Ar‐*H*), 4.31 (br, 2H, ‐C *H*_2_NH‐). ^13^C NMR (101 MHz, CDCl_3_) δ 146.3, 137.5, 133.1, 129.1, 128.9, 128.7, 122.5, 114.0 (Ar‐*C*), 47.69 (–*C* H_2_NH).

##### 2.1.3.4 *N*‐(4‐Chlorobenzyl)−4‐methylaniline (**2d**)

Yield: 86%, m.p:. 62.4–63.1°C. ^1^H NMR (400 MHz, CDCl_3_) δ 7.33 (s, 4H, Ar‐*H*), 7.02 (d, *J* = 8.1 Hz, 2H, Ar‐*H*), 6.57 (d, *J* = 8.3 Hz, 2H, Ar‐*H*), 4.32 (br, 2H, –C *H*_2_NH–), 2.27 (s, 3H, C *H*_*3*_Ph–). ^13^C NMR (101 MHz, CDCl_3_) δ 145.5, 138.2, 132.8, 129.8, 128.7, 127.1, 113.1(Ar‐*C*), 48.0 (–*C* H_2_NH), 20.4(*C* H_3_Ph–).

##### 2.1.3.5 4‐Chloro‐*N*‐(4‐nitrobenzyl)aniline (**2e**)

Yield: 87%, m.p:. 98.4–99.7°C. ^1^H NMR (400 MHz, CDCl_3_) δ 8.22 (d, *J* = 8.6 Hz, 2H, Ar‐*H*), 7.54 (d, *J* = 8.6 Hz, 2H, Ar‐*H*), 7.13 (d, *J* = 8.8 Hz, 2H, Ar‐*H*), 6.53 (d, *J* = 8.8 Hz, 2H, Ar‐*H*), 4.48 (br, 3H, –C *H*_2_NH–). ^13^C NMR (101 MHz, CDCl_3_) δ 147.3, 146.9, 145.8, 129.3, 127.7, 124.0, 122.9, 114.1 (Ar‐*C*), 47.7 (–*C* H_2_NH).

##### 2.1.3.6 4‐Methyl‐*N‐(*4‐nitrobenzyl)aniline (**2 f**)

Yield: 87%, m.p:. 73.8–74.5°C. ^1^H NMR (400 MHz, CDCl_3_) δ 8.21 (d, *J* = 8.5 Hz, 2H, Ar‐*H*), 7.56 (d, *J* = 8.5 Hz, 2H, Ar‐*H*), 7.01 (d, *J* = 8.1 Hz, 2H, Ar‐*H*), 6.54 (d, *J* = 8.2 Hz, 2H, Ar‐*H*), 4.48 (br, 2H, C *H*_2_NH–), 2.27 (s, 3H, C *H*_*3*_Ph–). ^13^C NMR (101 MHz, CDCl_3_) δ 147.7, 147.2, 145.0, 129.9, 127.7, 123.9, 113.1 (Ar‐*C*), 48.0 (–*C* H_2_NH), 20.4(*C* H_3_Ph–).

##### 2.1.3.7 4‐Chloro‐*N*‐(4‐methoxybenzyl)aniline (**2g**)

Yield: 91%, m.p:. 65.9–66.7°C. ^1^H NMR (400 MHz, CDCl_3_) δ 7.30 (d, *J* = 8.4 Hz, 2H, Ar‐*H*), 7.14 (d, *J* = 8.7 Hz, 2H, Ar‐*H*), 6.91 (d, *J* = 8.5 Hz, 2H, Ar‐*H*), 6.58 (d, *J* = 8.7 Hz, 2H, Ar‐*H*), 4.25 (br, 2H, C *H*_2_NH–), 3.83 (s, 3H, –OC *H*_*3*_). ^13^C NMR (101 MHz, CDCl_3_) δ 159.0, 146.6, 130.8, 129.1, 128.8, 122.1, 114.1, 114.0 (Ar‐*C*), 55.33 (–O *C* H_3_), 47.91 (–*C* H_2_NH).

##### 2.1.3.8 *N*‐(4‐Methoxybenzyl)−4‐methylaniline (**2h**)

Yield: 95%, m.p:. 70.6–71.1°C. ^1^H NMR (400 MHz, CDCl_3_) δ 7.32 (d, *J* = 8.4 Hz, 2H, Ar‐*H*), 7.02 (d, *J* = 8.3 Hz, 2H, Ar‐*H*), 6.91 (d, *J* = 8.5 Hz, 2H, Ar‐*H*), 6.61 (d, *J* = 8.3 Hz, 2H, Ar‐*H*), 4.27 (br, 2H, C *H*_2_NH–), 3.84 (s, 3H, –OC *H*_*3*_), 2.28 (s, 3H, C *H*_*3*_Ph–). ^13^C NMR (101 MHz, CDCl_3_) δ 158.8, 146.0, 131.6, 129.8, 128.8, 126.8, 114.0, 113.1 (Ar‐*C*), 55.3 (–O *C* H_3_), 48.2 (–*C* H_2_NH), 20.4 (*C* H_3_Ph–).

##### 2.1.3.9 *N*‐(4‐Bromobenzyl)−2‐(1*H*‐indol‐3‐yl)ethan‐1‐amine (**2i**)

Yield: 63%, m.p:. 86.2–86.8°C. ^1^H NMR (400 MHz, DMSO‐d_6_) δ 10.78 (s, 1H, indole NH), 7.48 (dd, *J* = 6.5, 1.9 Hz, 3H, Ar‐*H*), 7.31 (dd, *J* = 14.3, 8.2 Hz, 3H, Ar‐*H*), 7.13 (s, 1H, Ar‐*H*), 7.08–7.03 (m, 1H, Ar‐*H*), 6.99–6.94 (m, 1H, Ar‐*H*), 3.72 (d, *J* = 6.3 Hz, 2H, C *H*_2_NH–), 2.89–2.81 (m, 2H, C *H*_2_‐CH_2_), 2.79 (t, *J* = 6.6 Hz, 2H, CH_2_‐C *H*_2_), 2.08 (t, *J* = 6.5 Hz, 1H, NH). ^13^C NMR (101 MHz, DMSO‐d6) δ 141.1, 136.7, 131.4, 130.6, 127.7, 123.0, 121.3, 119.8, 118.8, 118.6, 113.1, 111.8, (Ar‐*C*), 52.6 (–*C* H_2_NH) 50.0 (–*C* H_2_NH), 26.0 (*C* H_2_‐CH_2_).

#### 2.1.4 General Procedure for the Preparation of Sulfonamide (4a–i)

An equimolar mixture of compounds (**2a**–**i**) and sodium carbonate in a THF/H_2_O (1:1) was stirred for 30 min at room temperature. After which, a solution of methylphenylsulfonyl chloride (0.80 mmol) in THF was added dropwise. Upon the completion of the reaction after 24–48 h as monitored by TLC, the solvent was removed under reduced pressure to afford an oily residue, which was acidified with 20% aqueous HCl. The resulting solid was filtered and recrystallized from ethanol to afford **4a**–**i** [13].

##### 2.1.4.1 *N*‐(4‐Bromobenzyl)‐*N*‐(4‐chlorophenyl)−4‐methylbenzenesulfonamide (**4a**)

Yield: 67%, m.p:. 264.1–264.9°C. IR_(KBr)_(cm^−1^): 2908 (C‐H Aliphatic); 1378 (SO_2_‐N); 721 (C‐Cl); 682 (C‐Br). ^1^H NMR (400 MHz, DMSO‐d_6_) δ 7.52 (dd, *J* = 10.1, 8.3 Hz, 4H, Ar‐*H*), 7.31 (d, *J* = 8.2 Hz, 2H, Ar‐*H*), 7.14 (d, *J* = 8.2 Hz, 4H, Ar‐*H*), 6.69 (d, *J* = 8.6 Hz, 2H, Ar‐*H*), 4.29 (s, 2H, –C *H*_2_NH–), 2.30 (s, 3H, C *H*_*3*_Ph–). ^13^C NMR (101 MHz, DMSO‐d6) δ 145.7, 138.4, 131.7, 130.3, 129.2, 128.6, 126.0, 120.6, 115.9 (Ar‐*C*), 47.3 (*C* H_2_NH–), 21.3 (*C* H_3_Ph–). HRMS (ESI^+^, FTMS) *m*/*z* for C_20_H_17_BrClNO_2_S [M]^+^ calcd. 448. 9852, found 448.2414 [M]^+^.

##### 2.1.4.2 *N*‐(4‐Bromobenzyl)−4‐methyl‐*N*‐(*p*‐tolyl)benzenesulfonamide (**4b**)

Yield: 76%, m.p:. 238.0–238.7°C. IR_(KBr)_(cm^−1^) 2962 (C‐H Aliphatic); 1343 (SO_2_‐N); 683 (C‐Br). ^1^H NMR (400 MHz, DMSO‐d_6_) δ 7.53 (d, *J* = 8.1 Hz, 2H, Ar‐*H*), 7.44 (dd, *J* = 11.9, 8.3 Hz, 4H, Ar‐*H*), 7.22 (d, *J* = 8.2 Hz, 2H, Ar‐*H*), 7.06 (d, *J* = 8.1 Hz, 2H, Ar‐*H*), 6.92 (d, *J* = 8.2 Hz, 2H, Ar‐*H*), 4.73 (s, 2H, C *H*_2_NH–), 2.42 (s, 3H, C *H*_*3*_Ph–), 2.22 (s, H, C *H*_*3*_Ph–). ^13^C NMR (101 MHz, DMSO‐d6) δ 144.1, 137.6, 136.4, 136.3, 135.3, 131.7, 130.8, 130.3, 129.9, 128.6, 127.9, 121.0 (Ar‐*C*), 53.3 (*C* H_2_NH–), 21.5 (*C* H_3_Ph–), 21.0 (*C* H_3_Ph–). HRMS (ESI^+^, FTMS) *m*/*z* for C_21_H_20_BrNO_2_S [M]^+^ calcd. 429.0398, found 429.2409 [M]^+^.

##### 2.1.4.3 *N*‐(4‐Chlorobenzyl)‐*N*‐(4‐chlorophenyl)−4‐methylbenzenesulfonamide (**4c**)

Yield: 82%, m.p:. 243.8‐244.6°C. IR _(KBr)_(cm^−1^) 2995 (C‐H Aliphatic); 1380 (SO_2_‐N); 746, 797 (2C‐Cl). ^1^H NMR (400 MHz, DMSO‐d_6_) δ 7.50 (d, *J* = 8.0 Hz, 2H, Ar‐*H*), 7.43 – 7.30 (m, 4H, Ar‐*H*), 7.14 (d, *J* = 8.4 Hz, 4H, Ar‐*H*), 6.69 (d, *J* = 8.7 Hz, 2H, Ar‐*H*), 4.31 (s, 2H, –C *H*_2_NH–), 2.30 (s, 3H, C *H*_*3*_Ph–). ^13^C NMR (101 MHz, DMSO‐d6) δ 145.8, 138.4, 132.1, 129.9, 129.2, 128.8, 128.6, 126.0 (Ar‐*C*), 47.19 (*C* H_2_NH–), 21.27 (*C* H_3_Ph–). HRMS (ESI^+^, FTMS) m/z**:** [M]^+^ calcd for C_20_H_17_Cl_2_NO_2_S: 405,0357; found: 405.1320 [M]^+^.

##### 2.1.4.4 *N*‐(4‐Chlorobenzyl)−4‐methyl‐*N*‐(*p*‐tolyl)benzenesulfonamide (**4d**)

Yield: 63%, m.p:. 243.3–243.8°C. IR_(KBr)_ (cm^−1^) 2578 (C‐H Aliphatic); 1371 (SO_2_‐N); 747 (C‐Cl). ^1^H NMR (400 MHz, DMSO‐d_6_) δ 7.50 (d, *J* = 8.0 Hz, 2H, Ar‐*H*), 7.48–7.40 (m, 5H, Ar‐*H*), 7.19 (d, *J* = 8.0 Hz, 2H, Ar‐*H*), 7.13 (d, *J* = 7.9 Hz, 2H, Ar‐*H*), 7.09 (d, *J* = 7.1 Hz, 1H, Ar‐*H*), 4.48 (s, 2H, –C *H*_2_NH–), 2.30 (s, 3H, C *H*_*3*_Ph–), 2.27 (s, 3H, C *H*_*3*_Ph–). ^13^C NMR (101 MHz, DMSO‐d6) δ 145.9, 138.3, 133.5, 131.8, 130.4, 129.0, 128.6, 126.0 (Ar‐*C*), 40.6 (*C* H_2_NH–), 21.3 (*C* H_3_Ph–), 20.9 (*C* H_3_Ph–). HRMS (ESI^+^, FTMS) m/z: [M]^+^ calcd for C_21_H_20_ClNO_2_S: 385,0903; found: 385.2114 [M]^+^.

##### 2.1.4.5 *N*‐(4‐Chlorophenyl)−4‐methyl‐*N*‐(4‐nitrobenzyl)benzenesulfonamide (**4e**)

Yield: 68.03%, m.p:. 247.9–248.6°C. IR_(KBr)_ (cm^−1^) 2720 (C‐H Aliphatic); 1525 (NO_2_); 1370 (SO_2_‐N); 748 (C‐Cl). ^1^H NMR (400 MHz, DMSO‐d_6_) δ 8.21 (d, *J* = 8.4 Hz, 2H, Ar‐*H*), 7.61 (d, *J* = 8.3 Hz, 2H, Ar‐*H*), 7.50 (d, *J* = 7.8 Hz, 2H, Ar‐*H*), 7.14 (d, *J* = 7.7 Hz, 2H, Ar‐*H*), 7.09 (d, *J* = 8.6 Hz, 2H, Ar‐*H*), 6.58 (d, *J* = 8.6 Hz, 2H, Ar‐*H*), 4.44 (s, 2H, C *H*_2_NH–), 2.30 (s, 3H, C *H*_*3*_Ph–). ^13^C NMR (101 MHz, DMSO‐d6) δ 148.8, 147.2, 147.0, 145.9, 138.3, 129.1, 128.6, 128.6, 126.0, 124.0, 120.3, 114.5 (Ar‐*C*), 46.4 (*C* H_2_NH–), 21.2 (*C* H_3_Ph–). HRMS (ESI^+^, FTMS) m/z: [M]^+^ calcd for C_20_H_17_ClN_2_O_4_S: 416,0598; found: 416.2943 [M]^+^.

##### 2.1.4.6 4‐Methyl‐*N*‐(4‐nitrobenzyl)‐*N*‐(*p*‐tolyl)benzenesulfonamide (**4f**)

Yield: 69%, m.p:. 266.3–266.7°C. IR_(KBr)_ (cm^−1^) 2919 (C‐H Aliphatic); 1518 (NO_2_); 1343 (SO_2_‐N). ^1^H NMR (400 MHz, DMSO‐d_6_) δ 8.23 (d, *J* = 8.5 Hz, 2H, Ar‐*H*), 7.67 (d, *J* = 8.5 Hz, 2H, Ar‐*H*), 7.50 (d, *J* = 7.9 Hz, 2H, Ar‐*H*), 7.12 (dd, *J* = 15.6, 8.0 Hz, 4H, Ar‐*H*), 6.93 (d, *J* = 7.3 Hz, 2H, Ar‐*H*), 4.57 (s, 2H, C *H*_2_NH–), 2.30 (s, 3H, C *H*_*3*_Ph–), 2.23 (s, 3H, C *H*_*3*_Ph–).^13^C NMR (101 MHz, DMSO‐d6) δ 147.5, 145.8, 138.4, 130.3, 128.6, 126.0, 124.0 (Ar‐*C*), 40.7 (*C* H_2_NH–), 21.3 (*C* H_3_Ph–), 20.8 (*C* H_3_Ph–). HRMS (ESI^+^, FTMS) m/z: [M]^+^ calcd for C_21_H_20_N_2_O_4_S: 396,1144; found: 396.6066 [M]^+^.

##### 2.1.4.7 *N*‐(4‐Chlorophenyl)‐*N*‐(4‐methoxybenzyl)−4‐methylbenzenesulfonamide (**4g**)

Yield: 70%. m.p:. 222.0–222.7°C. IR_(KBr)_ (cm^−1^) 2950 (C‐HAliphatic); 1364 (SO_2_‐N); 1034 (OCH_3_). ^1^H NMR (400 MHz, DMSO‐d_6_) δ 7.50 (d, *J* = 7.9 Hz, 2H, Ar‐*H*), 7.28 (dd, *J* = 16.8, 8.4 Hz, 4H,) Ar‐*H*, 7.14 (d, *J* = 7.8 Hz, 2H, Ar‐*H*), 6.90 (t, *J* = 7.2 Hz, 4H, Ar‐*H*), 4.30 (s, 2H, C *H*_2_NH–), 3.74 (s, 3H, –OC *H*_*3*_), 2.30 (s, 3H, C *H*_*3*_Ph–). ^13^C NMR (101 MHz, DMSO‐d6) δ 159.3, 145.8, 138.3, 130.2, 129.4, 128.6, 126.0, 114.3 (Ar‐*C*), 55.6 (–O *C* H_3_), 40.6 (*C* H_2_NH–), 21.3 (*C* H_3_Ph–). HRMS (ESI^+^, FTMS) m/z: [M]^+^ calcd for C_21_H_20_ClNO_3_S: 401,0852; found: 401.6417 [M]^+^.

##### 2.1.4.8 *N*‐(4‐Methoxybenzyl)−4‐methyl‐*N*‐(*p*‐tolyl)benzenesulfonamide (**4h**)

Yield: 56%, m.p:. 135.8–136.6°C. IR_(KBr)_ (cm^−1^) 2920 (C‐HAliphatic); 1361 (SO_2_‐N); 1028 (OCH_3_). ^1^H NMR (400 MHz, DMSO‐d_6_) δ 7.53 (d, *J* = 8.1 Hz, 2H, Ar‐*H*), 7.42 (d, *J* = 8.0 Hz, 2H, Ar‐*H*), 7.14 (d, *J* = 8.4 Hz, 2H, Ar‐*H*), 7.04 (d, *J* = 8.1 Hz, 2H, Ar‐*H*), 6.88 (d, *J* = 8.1 Hz, 2H, Ar‐*H*), 6.80 (d, *J* = 8.5 Hz, 2H, Ar‐*H*), 4.67 (s, 2H, C *H*_2_NH–), 3.68 (s, 3H, –OC *H*_*3*_), 2.43 (s, 3H, C *H*_*3*_Ph–), 2.22 (s, 3H, C *H*_*3*_Ph–). ^13^C NMR (101 MHz, DMSO‐d6) δ 159.0, 143.9, 137.4, 136.4, 135.6, 130.2, 129.9, 129.8, 128.7, 128.5, 127.8, 114.1 (Ar‐*C*), 55.4 (–O *C* H_3_), 53.4 (*C* H_2_NH–), 21.5 (*C* H_3_Ph–), 20.9 (*C* H_3_Ph–). HRMS (ESI^+^, FTMS) m/z:[M]^+^ calcd for C_22_H_23_NO_3_S:381.1399; found: 381.1386 [M]^+^.

##### 2.1.4.9 *N*‐(2‐(1*H*‐indol‐3‐yl)ethyl)‐*N*‐(4‐bromobenzyl)−4‐methylbenzenesulfonamide (**4i**)

Yield: 60%, m.p:. 148.2–148.6°C. IR _(KBr)_ (cm^−1^) 3424 (N‐H indole); 2925 (CH‐Aliphatic);1347 (SO_2_‐N). ^1^H NMR (400 MHz, DMSO‐d_6_) δ 10.81 (s, 1H, indole NH), 7.77 (d, *J* = 8.2 Hz, 2H, Ar‐*H*), 7.47–7.38 (m, 6H, Ar‐*H*), 7.31 (d, *J* = 8.1 Hz, 1H, Ar‐*H*), 7.21 (d, *J* = 7.9 Hz, 1H, Ar‐*H*), 7.09–6.99 (m, 2H, Ar‐*H*), 6.93 (t, *J* = 7.4 Hz, 1H, Ar‐*H*), 4.39 (s, 2H, C *H*_2_NH–), 3.27 (dd, *J* = 9.6, 6.7 Hz, 2H, CH_2_NH), 2.68 (dd, *J* = 9.5, 6.7 Hz, 2H, CH_2_), 2.41 (s, 3H, C *H*_*3*_Ph–). ^13^C NMR (101 MHz, DMSO‐d6) δ 143.8, 136.9, 136.6, 132.7, 130.5, 130.4, 128.9, 127.4, 127.2, 123.37, 121.4, 118.8, 118.3, 111.9, 110.9 (Ar‐*C*), 51.3 (–*C* H_2_NH), 49.4 (–*C* H_2_NH), 25.0 (*C* H_2_‐CH_2_), 21.5 (*C* H_3_Ph–). HRMS (ESI^+^, FTMS) m/z: [M]^+^ calcd for C_24_H_23_BrN_2_O_2_S: 482,0664; found [M + 1]^+^ **:** 483.0735 [M]^+^.

## 3 Antioxidant Activity

The antioxidant activity was evaluated using the DPPH (2,2‐diphenyl‐1‐picrylhydrazyl) free radical scavenging assay, following a slightly modified previously reported procedure [14, 15]. The radical scavenging capacity of the synthesized compounds was determined using DPPH. Solutions of the compounds were prepared in DMSO‐d6 at five different concentrations (12.5, 25.0, 37.5, 62.5, and 125 μg/mL). Each solution was incubated for 20 min at room temperature. After incubation, the absorbance of each sample was measured at 517 nm using a UV–Vis spectrophotometer. The percentage of DPPH radical scavenging activity was calculated using the equation shown below.

$$\text{Scavenging effect}\;\left(\%\right)=\frac{\text{Control absorbtion}-\text{Test absorbance}}{\text{Control absorbtion}}\times 100$$

### 3.1 Cytotoxicity Studies

#### 3.1.1 Human Cancer Cell Lines and Culture Conditions

The human ovarian cancer cell line A2780 (ovarian carcinoma) and prostate cancer cell line LNCaP (Lymph Node Carcinoma of the Prostate) LNCaP used in this study were sourced from ATCC and cultured in RPMI‐1640 medium with 10% FBS and 1% penicillin/streptomycin at 37°C in a 5% CO_2_ humidified incubator. Experiments were conducted during the logarithmic growth phase using MTT reagent and analytical grade chemicals [16, 17].

*MTT Assay*: The anticancer activity of the synthesized compounds (**4a**–**i**) was performed against the A2780 and the LNCaP cell lines using the MTT (3‐(4,5‐dimethylthiazol‐2‐yl)‐2,5‐diphenyltetrazolium bromide) reduction assay. The MTT colorimetric assay was conducted on 96‐well cell culture plates at a density of 1 × 10^3^ per well (final volume: 100 μL), The plates were incubated for 24 h, the medium was replaced with fresh medium (100 µL) containing varying concentrations of the studied compounds (0.1, 1, 10, and 100 μg/mL). After a 24‐h a volume of MTT Solution (0.5 mg/mL, 100 µL), was introduced into each well and subjected to an additional 3 h incubation at a temperature of 37°C. Next, the MTT solution was aspirated, 100 μL of dimethyl sulfoxide (DMSO‐d6) solvent was added to dissolve the resulting formazan crystals and absorbance was measured at 570 nm using a microplate reader (BioTek Synergy HT) [18, 19, 20].

### 3.2 Molecular Docking

**Figure 1:**
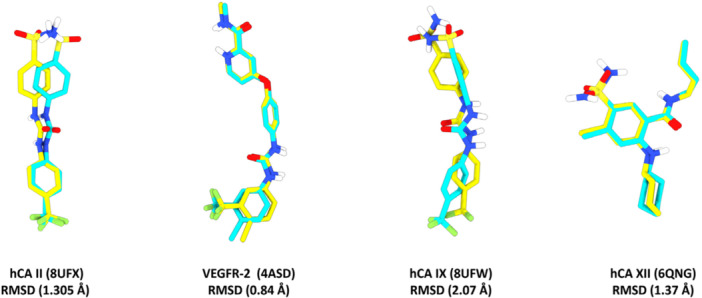
Validation of the docking protocol by re‐docking the co‐crystallized ligands into the active sites of hCA II (PDB: 8UFX), VEGFR‐2 (PDB: 4ASD), hCA IX‐mimic (PDB: 8UFW), and hCA XII (PDB: 6QNG).

Crystal structures of human carbonic anhydrase II (hCA II, PDB: 8UFX), vascular endothelial growth factor receptor‐2 (VEGFR‐2, PDB: 4ASD), human carbonic anhydrase IX (hCA IX, PDB: 8UFW), and human carbonic anhydrase XII (hCA XII, PDB: 6QNG) were retrieved from the Protein Data Bank. Protein structures were prepared by removing crystallographic water molecules and co‐crystallized ligands prior to docking. The chemical structures of the investigated compounds were constructed and geometry‐optimized using Avogadro and the optimized conformations were used for subsequent docking studies. Protein and ligand preparation was carried out in AutoDockTools v1.5.7 [21] by adding polar hydrogens, assigning appropriate atom types, and calculating Gasteiger charges, and the prepared structures were saved in PDBQT format. Docking simulations were performed using AutoDock Vina v1.2.7 with an exhaustiveness value of 40 and 8 output poses [22]. The docking grid box dimensions were set to 20 × 20 × 20 Å for all targets. Grid centers were defined at (15.46, 20.46, 12.92 Å) for hCA II, (−24.62, −0.38, −10.92 Å) for VEGFR‐2, (15.97, 6.96, 13.60 Å) for hCA IX, and (17.91, 24.93, −12.47 Å) for hCA XII, based on the corresponding native ligand‐binding sites. The docking protocol was validated by re‐docking the co‐crystallized ligands into their respective binding pockets, yielding RMSD values of 1.305 Å for hCA II, 0.84 Å for VEGFR‐2, 2.07 Å for hCA IX, and 1.37 Å for hCA XII, indicating acceptable agreement between the predicted and experimental binding modes (Figure 1). Docked conformations and protein‐ligand interactions were visualized and analyzed using Discovery Studio Visualizer (Dassault Systèmes BIOVIA) and UCSF Chimera [23].

## 4 Results and Discussion

### 4.1 Chemistry

**Scheme 1:**
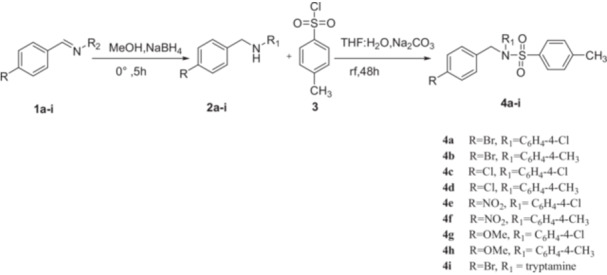
General scheme for the synthesis of sulfonamide (**4a**–**i**).

The synthetic route to the target sulfonamide derivatives **4a**–**i** is outlined in Scheme 1. The initial step involved the preparation of a series of Schiff bases from the condensation of appropriately substituted aldehydes with primary amines. Subsequent reduction of the Schiff bases with sodium borohydride (NaBH4) in methanol at 0°C for 5 h afforded the corresponding secondary amines **2a**–**i** in good yields. The successful reduction was confirmed by ^1^H and ^13^C NMR spectroscopy. In the ^1^H NMR spectra of the reduced intermediates, a diagnostic signal attributable to the *N*–CH_2_ methylene protons was observed in the aliphatic region (δ 3.72–4.48 ppm), whereas the characteristic imine singlet (δ 8.00–9.00 ppm) was conspicuously absent, thereby confirming the complete reduction of the azomethine bond. The secondary amines **2a**–**i** was subsequently subjected to *N*‐sulfonylation with methanesulfonyl chloride in the presence of sodium carbonate as a base, employing a THF/H_2_O biphasic solvent system at ambient temperature for 24–48 h. This mild reaction protocol furnished the desired sulfonamide derivatives **4a**–**i** in satisfactory yields.

The structures of all target compounds were rigorously established through a combination of FT‐IR, ^1^H NMR, ^13^C NMR, and HRMS analyses. The FT‐IR spectra of the final products exhibited characteristic asymmetric and symmetric S = O stretching vibrations in the 1342–1380 cm^−1^ region, unequivocally confirming the successful incorporation of the sulfonamide functionality. In the representative ^1^H NMR spectrum of compound **4b**, a diagnostic singlet at δ 2.30 ppm was assigned to the N–SO_2_C*H*_3_ methyl protons, while the aromatic protons resonated as doublets and doublets of doublets in the δ 6.69–7.52 ppm region. The ^13^C NMR spectrum revealed aromatic carbon signals spanning δ 115.9–145.9 ppm, with the aliphatic region displaying characteristic resonances at δ 47.3 ppm (N–CH_2_) and δ 21.3 ppm (Ar–CH_3_). High‐resolution mass spectrometric analysis provided further structural confirmation; for instance, compound **4b** displayed a molecular ion peak at m/z 448.2414 [M]^+^, which was in excellent agreement with the calculated molecular formula. All spectroscopic data for the remaining compounds were fully consistent with the proposed structures.

### 4.2 Antioxidant Activity

The antioxidant potential of the synthesized sulfonamide derivatives **4a**–**i** was evaluated by the 2,2‐diphenyl‐1‐picrylhydrazyl (DPPH) free radical scavenging assay, a widely employed method for assessing the hydrogen‐donating ability of potential antioxidant, Butylated hydroxytoluene (BHT) was employed as the reference standard [14, 15], and the results are summarized in Table 1.

**Table 1:** Antioxidant Activities of the sulfonamides derivatives, 4a–i.

Compound	12.5 μg/mL % activity	25 μg/mL % activity	37.5 μg/mL % activity	62.5 μg/mL % activity	125 μg/mL % activity
**4a**	31.45	38.25	44.45	48.78	49.23
**4b**	24.33	35.16	45.63	48.55	48.93
**4c**	21.26	34.14	38.54	43.56	45.45
**4d**	54.40	60.25	67.20	72.56	75.45
**4e**	24.33	31.34	35.548	40.18	44.18
**4f**	23.18	29.65	38.65	44.18	46.11
**4g**	21.33	26.65	33.17	36.00	40.34
**4h**	23.56	25.65	32.34	38.45	40.18
**4i**	20.26	24.45	29.90	36.88	39.80
**BHT**	59.98	62.67	65.32	69.82	76.34

Among the tested compounds, **4d** exhibited remarkably potent antioxidant activity across all concentrations examined. Notably, at 37.5 and 62.5 µg/mL, compound **4d** displayed superior radical scavenging capacity (67.20% and 72.56%, respectively) compared to BHT (65.32% and 69.82%, respectively). At the remaining concentrations, the antioxidant activity of **4d** remained closely comparable to that of BHT, further underscoring its promising antioxidant potential. Compounds **4a**, **4b**, **4c**, **4e**, **4f**, **4g**, and **4h** exhibited moderate antioxidant activity, particularly at higher concentrations, whereas compound **4i** demonstrated the lowest activity among all derivatives evaluated.

The observed structure–activity relationships reveal that the nature and position of substituents on the aromatic ring exert a profound influence on antioxidant efficacy. Compound **4d**, bearing one chlorine atom and two methyl groups, benefits from a synergistic electronic interplay: the electron‐withdrawing chlorine substituent contributes to the stabilization of the resultant radical species following hydrogen atom transfer, while the electron‐donating methyl groups enhance the electron density of the aromatic system, thereby facilitating radical scavenging. This favorable combination of electronic effects accounts for the superior antioxidant activity of **4d**. The remaining compounds, functionalized with nitro, methoxy, or bromo substituents, exhibited moderate antioxidant activity, which can be rationalized on the basis of the steric and electronic characteristics of these groups.

### 4.3 Cytotoxicity Studies

The in vitro cytotoxic potential of the sulfonamide derivatives **4a**–**i** was assessed against two human cancer cell lines: A2780 (ovarian carcinoma) and LNCaP (lymph node carcinoma of the prostate) using the MTT colorimetric assay, with docetaxel serving as a positive control. Cells were incubated with the test compounds at concentrations ranging from 0.1 to 100 µg/mL for 24 h, and cell viability was determined. The log IC_50_ values, derived from dose–response curves, are presented in Table 2, while detailed cell viability data are provided in Tables 3 and 4.

**Table 2:** Log IC_50_ concentrations calculated for LNCaP, A2780 cell lines for the new sulfonamides derviatives (4a–i).

Compound	LNCaP LogIC_50_	A2780 LogIC_50_
**4a**	2.096	2.17
**4b**	1.835	2.223
**4c**	2,072	1.835
**4d**	2.065	1.814
**4e**	1.953	2.072
**4f**	2.055	2.15
**4g**	2.01	1.089
**4h**	1.343	1.585
**4i**	1.686	1.164
**Docetaxel**	−0.7728	−0.7944

As shown in Table 2, compound **4h** demonstrated the most potent cytotoxic activity against LNCaP cells, with a log IC_50_ = 1.343, followed by compound **4i** (log IC_50_ = 1.686). The remaining compounds displayed moderate cytotoxicity against LNCaP cells, with log IC_50_ values ranging from 1.835 to 2.096. Against the A2780 cell line, compounds **4g**, **4i**, and **4h** exhibited the highest cytotoxic potency, with log IC_50_ = 1.089, 1.164, and 1.585, respectively, while the remaining compounds showed moderate activity (log IC_50_ range: 1.814–2.223).

The structure–activity relationship analysis suggests that the presence of the methoxy substituent in compounds **4g** and **4h** plays a critical role in enhancing cytotoxic activity. The methoxy group, acting as an electron‐donating substituent, increases the electron density of the aromatic ring, thereby potentially facilitating interactions with key biological targets. Furthermore, the enhanced lipophilicity conferred by the methoxy group may improve cellular membrane permeability, leading to greater intracellular accumulation of the active compounds [24].

The cell viability data for the A2780 cell line (Table 3) revealed that compounds **4c**, **4d**, and **4g** exhibited statistically significant cytotoxic activity (*p* < 0.05) across all tested concentrations (0.1–100 µg/mL), indicating a broad effective dose range. The remaining derivatives displayed concentration‐dependent cytotoxicity, with significant effects predominantly observed at higher concentrations (10 and 100 µg/mL), with the exception of compound **4f**, which exhibited significant cytotoxicity only at 100 µg/mL.

**Table 3:** A2780 Cell viability (**%**) results with compounds 4a–i.

A2780						
**Compound**	Control	DMSO‐d6	0.1 μg/mL	1 μg/mL	10 μg/mL	100 μg/mL
**4a**	100 ± 10.21	94.02 ± 10.81	78.28 ± 8.56	70.68 ± 7.36*	70.07 ± 6.20*	66.35 ± 7.21*
**4b**	100 ± 10.21	94.02 ± 10.81	75.94 ± 8.06	74.16 ± 9.933	69.90 ± 5.09*	68.84 ± 6.14*
**4c**	100 ± 10.21	94.02 ± 10.81	64.17 ± 7.21*	63.60 ± 5.08*	58.43 ± 6.08*	56.20 ± 5.39*
**4d**	100 ± 10.21	94.02 ± 10.81	59.88 ± 6.45*	60.05 ± 6.94*	59.44 ± 5.53*	55.28 ± 4.64*
**4e**	100 ± 10.21	94.02 ± 10.81	82.15 ± 10.82	76.62 ± 9.28	64.21 ± 5.75*	63.05 ± 7.02*
**4f**	100 ± 10.21	94.02 ± 10.81	87.24 ± 9.68	79.94 ± 10.03	81.66 ± 9.37	62.14 ± 6.88*
**4g**	100 ± 10.21	94.02 ± 10.81	66.55 ± 7.12*	59.48 ± 6.02*	50.13 ± 6.21*	49.84 ± 5.76*
**4h**	100 ± 10.21	94.02 ± 10.81	78.29 ± 9.95	72.72 ± 9.86	65.43 ± 6.46*	42.48 ± 4.22*
**4i**	100 ± 10.21	94.02 ± 10.81	81.88 ± 10.83	59.36 ± 6.09*	52.07 ± 5.13*	48.04 ± 5.32*
**Docetaxel**	100 ± 10.21	94.02 ± 10.81	52.71 ± 6.13*	29.86 ± 2.39*	14.12 ± 1.16*	1.07 ± 0.23*

*Note:* The cell viability values obtained from the sulfonamides derivatives are shown as a percentage of absorbance (MTT) compared to the control. Each data point reflects the average of 10 viability measurements.

* *p* < 0.05.

For the LNCaP cell line (Table 4), the sulfonamide derivatives generally exhibited significant cytotoxicity only at higher concentrations (10 and 100 µg/mL), suggesting a somewhat lower sensitivity of this cell line compared to A2780. Again, compound **4f** demonstrated significant activity exclusively at 100 µg/mL. Collectively, these results indicate that the A2780 ovarian carcinoma cell line is generally more susceptible to the cytotoxic effects of the synthesized sulfonamide derivatives than the LNCaP prostate cancer cell line.

**Table 4:** LNCaP Cell viability (%) results with compounds 4a–i.

LNCaP						
**Compound**	Control	DMSO‐d6	0.1 μg/mL	1 μg/mL	10 μg/mL	100 μg/mL
**4a**	100 ± 8.09	95.58 ± 9.75	98.51 ± 10.86	95.97 ± 12.25	68.07 ± 7.25*	62.43 ± 5.69*
**4b**	100 ± 8.09	95.58 ± 9.75	96.27 ± 10.43	93.08 ± 8.06	59.06 ± 4.49*	53.96 ± 4.23*
**4c**	100 ± 8.09	95.58 ± 9.75	87.25 ± 9.86	92.70 ± 10.71	70.75 ± 6.02*	60.63 ± 5.62*
**4d**	100 ± 8.09	95.58 ± 9.75	87.48 ± 9.09	84.63 ± 9.13	63.99 ± 8.35*	62.51 ± 6.05*
**4e**	100 ± 8.09	95.58 ± 9.75	95.67 ± 11.05	85.41 ± 12.02	62.43 ± 6.18*	57.89 ± 5.34*
**4f**	100 ± 8.09	95.58 ± 9.75	92.64 ± 12.89	90.45 ± 10.47	75.27 ± 9.73	58.45 ± 7.58*
**4g**	100 ± 8.09	95.58 ± 9.75	89.60 ± 10.21	93.17 ± 7.27	69.85 ± 6.64*	57.71 ± 5.35*
**4h**	100 ± 8.09	95.58 ± 9.75	97.91 ± 8.16	85.47 ± 8.19	64.89 ± 7.21*	26.62 ± 4.12*
**4i**	100 ± 8.09	95.58 ± 9.75	94.86 ± 7.19	86.10 ± 9.51	64.50 ± 8.13*	45.63 ± 4.04*
**Docetaxel**	100 ± 9.43	91.36 ± 10.63	56.12 ± 5.24*	25.82 ± 2.96*	10.75 ± 1.24*	2.42 ± 0.42*

*Note:* The cell viability values obtained from the sulfonamides derviatives are shown as a percentage of absorbance (MTT) compared to the control. Each data point reflects the average of 10 viability measurements.

* *p* < 0.05.

### 4.4 Molecular Docking

In order to rationalize the promising anticancer activity of the most potent sulfonamide derivatives (**4g, 4h**, and **4i**), molecular docking studies were performed against VEGFR‐2, hCA II, hCA IX‐mimic, and hCA XII. These targets were selected because they represent two complementary pathways that are highly relevant to tumor progression, namely angiogenesis and pH regulation. VEGFR‐2 is a key driver of tumor angiogenesis and remains one of the most validated molecular targets in anticancer therapy, while sulfonamide‐based derivatives have shown considerable promise as VEGFR‐2 inhibitors and, in some cases, as multitarget agents [25, 26].

On the other hand, hCA IX and hCA XII are hypoxia‐associated tumor carbonic anhydrase isoforms that facilitate cancer cell adaptation to the acidic tumor microenvironment and are strongly implicated in invasion, metastasis, and treatment resistance [27, 28].

hCA II was additionally included as a reference isoform to compare ligand accommodation within the conserved carbonic anhydrase active site and to provide an indication of possible isoform selectivity among the tested compounds. Thus, the selected docking panel was considered suitable for exploring whether the observed biological activity of the synthesized sulfonamides could be associated with interference in VEGFR‐2‐driven angiogenic signaling and/or inhibition of tumor‐associated carbonic anhydrases.

The docking scores of the selected sulfonamide derivatives against VEGFR‐2, hCA II, hCA IX, and hCA XII are presented in Table 5. In the present docking set, the co‐crystallized ligands used for protocol validation were BAX for VEGFR‐2/4ASD, WJN for hCA II/8UFX, WJN for hCA IX‐mimic/8UFW, and benzenesulfonamide for hCA XII/6QNG. As shown in Table 5, compounds **4g**–**4i** displayed more favorable binding energies than the native ligand in hCA II, with values of −8.83, −8.94, and −9.44 kcal/mol, respectively, compared with −7.95 kcal/mol for the native ligand, suggesting a particularly strong affinity of these derivatives toward this isoform. Against hCA XII, compound **4i** also exhibited the most favorable score (−9.22 kcal/mol), surpassing the native ligand (−8.75 kcal/mol), whereas **4g** and **4h** showed slightly weaker but still comparable affinities (−8.15 and −8.17 kcal/mol). In the case of hCA IX, compound **4i** again emerged as the best derivative (−8.47 kcal/mol), with a value very close to that of the native ligand (−8.53 kcal/mol), while **4g** and **4h** showed only slightly lower binding affinities (−8.09 and −8.17 kcal/mol). By contrast, all tested compounds were markedly less favored in VEGFR‐2 than the co‐crystallized ligand, giving docking scores between −7.46 and −7.62 kcal/mol versus −12.03 kcal/mol for the native ligand. Overall, these findings indicate that the anticancer‐active sulfonamide derivatives, particularly compound **4i**, are more likely to exert their activity through preferential interaction with carbonic anhydrase isoforms, especially hCA II and hCA XII, rather than through strong VEGFR‐2 binding under the present docking conditions.

**Table 5:** Docking scores of the native ligands and the most active sulfonamide derivatives (4g–4i) against hCA II, VEGFR‐2, hCA IX‐mimic, and hCA XII, expressed as binding energy values (kcal/mol).

Ligand	Binding energy in kcal/mol			
	hCA II (8UFX)	VEGFR‐2 (4ASD)	hCA IX (8UFW)	hCA XII 6QNG)
Native	−7.95	−12.03	−8.53	−8.75
**4g**	−8.83	−7.46	−8.09	−8.15
**4h**	−8.94	−7.62	−8.17	−8.17
**4i**	−9.44	−7.52	−8.47	−9.22

**Figure 2:**
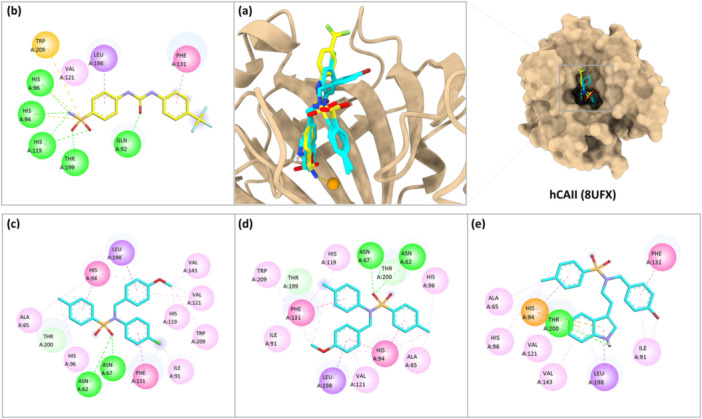
Binding mode analysis of the most active sulfonamide derivatives within the hCA II active site. (a) Superposition of the docked poses of compounds **4g**–**4i** with the co‐crystallized ligand inside the binding pocket of hCA II (PDB: 8UFX). (b) Two‐dimensional interaction diagram of the native ligand. (c–e) Two‐dimensional interaction diagrams of compounds **4g, 4h**, and **4i**, respectively.

To further elucidate the binding modes of the most active sulfonamide derivatives toward the studied enzymes, their interaction profiles were compared with those of the corresponding co‐crystallized ligands. For hCA II (Figure 2), the three docked compounds retained several of the key contacts observed for the native ligand, which supports a similar accommodation within the active site. The most consistent shared interactions involved Phe131, Leu198, and Val121, indicating preservation of the same hydrophobic recognition region. In addition, His94 and/or His96 were also maintained by the docked compounds, suggesting that they occupy the catalytic pocket in a manner comparable to the native ligand. Among the tested derivatives, **4h** showed the closest overlap with the native ligand, as it preserved the largest number of common contacts, including His94, His96, His119, Thr199, Phe131, Leu198, Val121, and Trp209. These interaction pattern indicates that compounds **4g**–**4i** bind hCA II through the same principal anchoring residues involved in native‐ligand recognition.

**Figure 3:**
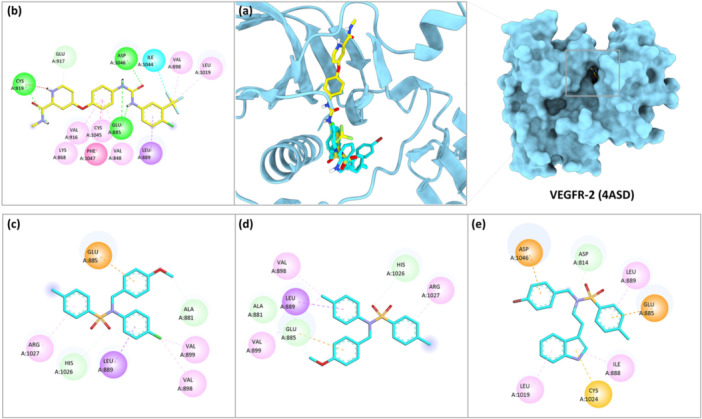
Binding mode analysis of the most active sulfonamide derivatives within the VEGFR‐2 active site. (a) Superposition of the docked poses of compounds **4g**–**4i** with the co‐crystallized ligand inside the binding pocket of VEGFR‐2 (PDB: 4ASD). (b) Two‐dimensional interaction diagram of the native ligand. (c–e) Two‐dimensional interaction diagrams of compounds **4g, 4h,** and **4i**, respectively.

For VEGFR‐2 (Figure 3), the docked compounds showed only limited overlap with the interaction pattern of the native ligand, in agreement with their lower docking scores. The main common contact retained by all three derivatives was Glu885, indicating that this residue represents the most conserved recognition point within the binding site. In addition, compounds **4g** and **4h** also shared interaction with Val898, while compound **4i** reproduced a broader part of the native binding environment through common contacts with Asp1046, Leu1019, and Glu885. Therefore, among the tested derivatives, **4i** displayed the closest interaction profile to the co‐crystallized ligand, although the overall similarity remained lower than that observed for the carbonic anhydrase targets.

**Figure 4:**
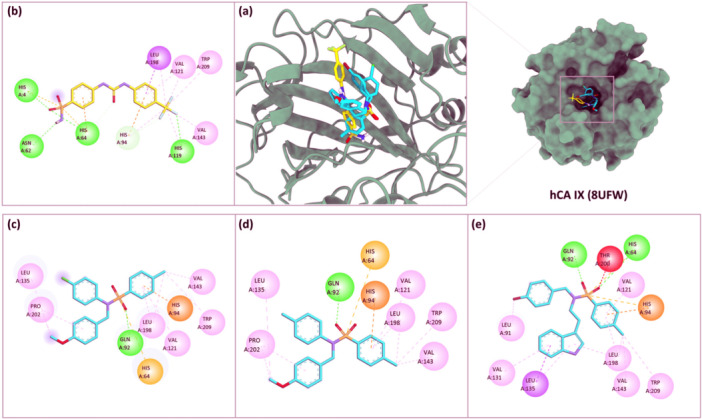
Binding mode analysis of the most active sulfonamide derivatives within the hCA IX active site. (a) Superposition of the docked poses of compounds **4g**–**4i** with the co‐crystallized ligand inside the binding pocket of hCA IX (PDB: 8UFW). (b) Two‐dimensional interaction diagram of the native ligand. (c–e) Two‐dimensional interaction diagrams of compounds **4g, 4h,** and **4i**, respectively.

For hCA IX (Figure 4), the docked sulfonamide derivatives reproduced several of the key contacts observed for the native ligand, supporting a comparable accommodation within the active site. The main common interactions were those with His64, His94, Val121, Leu198, Val143, and Trp209. Compound **4g** retained all of these shared contacts, while **4h** and **4i** also showed a close similarity to the native ligand by preserving the same principal interaction network within the binding pocket. Overall, the three compounds displayed a broadly similar binding pattern to that of the co‐crystallized ligand, indicating that they are well accommodated in the hCA IX active site through the same main anchoring residues.

**Figure 5:**
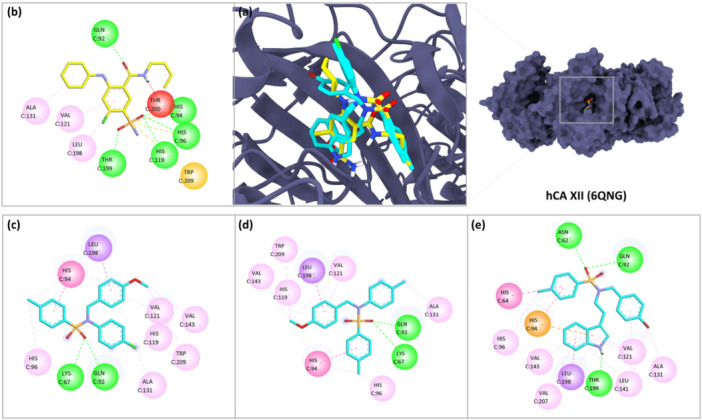
Binding mode analysis of the most active sulfonamide derivatives within the hCA XII active site. (a) Superposition of the docked poses of compounds **4g**–**4i** with the co‐crystallized ligand inside the binding pocket of hCA XII (PDB: 6QNG). (b) Two‐dimensional interaction diagram of the native ligand. (c–e) Two‐dimensional interaction diagrams of compounds **4g, 4h,** and **4i**, respectively.

Finally, for hCA XII (Figure 5), the docked sulfonamide derivatives retained several of the key contacts observed for the native ligand, indicating a closely related binding mode within the active site. The most consistently shared interactions involved Gln92, His94, His96, Leu198, Val121, and Ala131, which were preserved by the three tested compounds. In addition, **4g** and **4h** also maintained common contacts with His119 and Trp209, whereas **4i** reproduced the native binding pattern through shared interactions with Thr199 in addition to the conserved residues. Overall, the interaction profiles indicate that compounds **4g**–**4i** occupy the hCA XII binding pocket through the same principal anchoring residues involved in recognition of the co‐crystallized ligand.

Collectively, the docking results support a clearer preference of the selected sulfonamide derivatives for the carbonic anhydrase binding sites over VEGFR‐2. This conclusion is supported by both the docking scores and the interaction analysis. In the carbonic anhydrase isoforms, the docked compounds reproduced a larger portion of the native binding pattern and showed better spatial accommodation within the catalytic cavity, consistent with the known compatibility of sulfonamide derivatives with carbonic anhydrase active sites. In addition, part of this stabilization appears to arise from directional polar contacts consistent with hydrogen‐bonding interactions, together with hydrophobic fitting within the pocket. By contrast, although some relevant contacts were observed in VEGFR‐2, the compounds showed weaker binding energies and a more limited overlap with the interaction network of the co‐crystallized ligand, indicating less efficient occupation of the kinase active site under the present docking conditions. Taken together, these findings suggest that carbonic anhydrases, particularly hCA II, hCA IX, and hCA XII, are more plausible molecular targets than VEGFR‐2 for the most active sulfonamide derivatives, with compound **4i** showing the most favorable overall docking profile among the tested ligands.

## 5 Conclusion

In summary, a series of novel sulfonamide derivatives (**4a**–**i**) was successfully synthesized via a straightforward three‐step sequence involving Schiff base formation, sodium borohydride reduction, and *N*‐sulfonylation with methylphenylsulfonyl chloride. All target compounds were thoroughly characterized by FT‐IR, ^1^H NMR, ^13^C NMR, and HRMS analyses, which unequivocally confirmed their structures. Biological evaluation revealed that compound **4d** possesses outstanding antioxidant activity, surpassing the reference standard BHT at specific concentrations, attributable to the synergistic electronic effects of its chloro and methyl substituents. Cytotoxicity was screening against A2780 and LNCaP cancer cell lines and compounds **4g**, **4h**, and **4i** was identified as the most potent candidates, with **4h** exhibiting the lowest log IC_50_ value (1.343) against LNCaP cells. The methoxy substituent was identified as a key structural determinant of enhanced cytotoxic activity, likely through increased lipophilicity and electron donation facilitating target engagement. These findings highlight the potential of sulfonamide‐based scaffolds as dual antioxidant–anticancer agents and provide a foundation for further structural optimization and mechanistic investigation. Docking studies revealed that sulfonamide derivatives **4g**, **4h**, and **4i** are potential inhibitor against VEGFR‐2, hCA II, hCA IX‐mimic, and hCA XII.

## Author Contributions

**Ouissal Bouraoui:** investigation, writing – original draft. **Güldeniz Şekerci:** investigation. **Khaled Mesbah:** writing – review and editing. **James A. Ezugwu:** investigation, methodology, writing – review and editing. **Rachid Benkiniouar:** writing – review and editing. **Suat Tekin:** investigation, methodology. **Fatümetüzzehra Küçükbay:** investigation, methodology. **Houssem Boulebd:** data curation, software, methodology. **Hasan Küçükbay:** conceptualization, investigation, methodology, writing – review and editing, project administration, supervision.

## Conflicts of Interest

The authors declare no conflicts of interest.

## Supporting information

Supporting File

## Data Availability

The data that supports the findings of this study are available in the supporting material of this article.
