# Supplementary material for: Synthesis, Characterization, Antioxidant Evaluation, Cytotoxicity Studies, and Molecular Docking of Novel Sulfonamide Derivatives
Source: J Biochem Mol Toxicol. 2026 Jun 23;40(7):e70981. doi: 10.1002/jbt.70981 (PMC13288007; doi:10.1002/jbt.70981)
Supplement: Supplementary file 1 — Supporting File [file JBT-40-e70981-s001.pdf]

Supplementary Material

Synthesis, Characterization, Antioxidant Evaluation, Cytotoxicity Studies, and Molecular Docking of Novel Sulfonamide Derivatives

Ouissal Bouraoui[1,2], Güldeniz Şekerci[3], Khaled Mesbah[1], James A. Ezugwu[2,4] , Rachid Benkiniouar[1], Suat Tekin[3], Fatümetüzzehra Küçükbay[5], Houssem Boulebd[6], Hasan Küçükbay[2] *

[1]*Laboratory of Natural Products of Plant Origin and Organic Synthesis, Frères Mentouri University Constantine 1, Algeria*

[2] Department of Chemistry, Faculty of Arts and Sciences, İnönü University, 44280 Malatya, Turkey

[3] Department of Physiology, Faculty of Medicine, İnönü University, 44280 Malatya, Turkey

[4]*Department of Pure and Industrial Chemistry, University of Nigeria, Nsukka, 410001, Enugu State, Nigeria*

[5]İnonu University, Faculty of Pharmacy, Department of Basic Pharmaceutical Sciences, 44280 Malatya, Turkey

[6] Laboratory of Synthesis of Molecules With Biological Interest, Department of Chemistry, Faculty of Exact Sciences, University Frères Mentouri Constantine 1, Constantine, Algeria

Correspondence : *Email: hasan.kucukbay@inonu.edu.tr

_N_-(4-Bromobenzyl)-4-chloroaniline (2a)

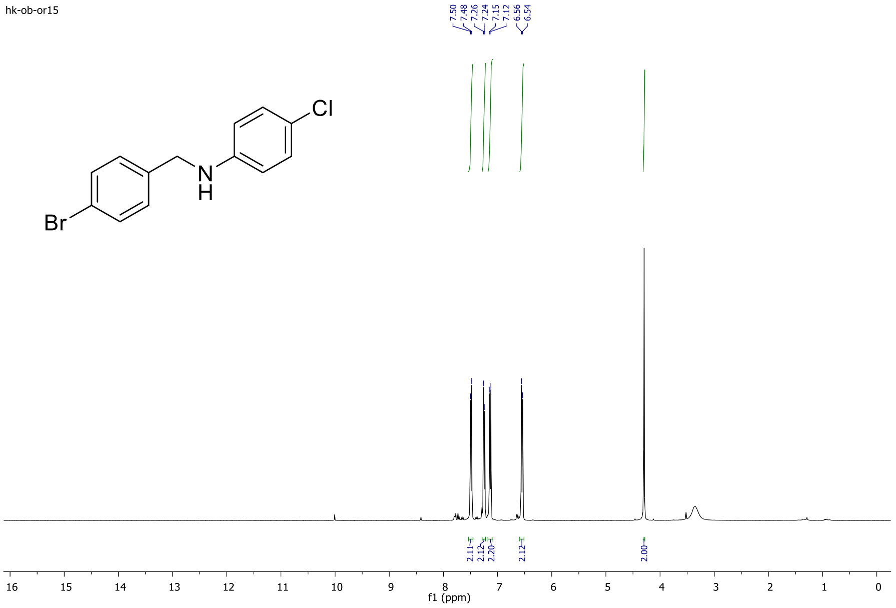

^{1}H NMR Spectrum of *N*-(4-bromobenzyl)-4-chloroaniline (**2a**) in $CDCl_3$

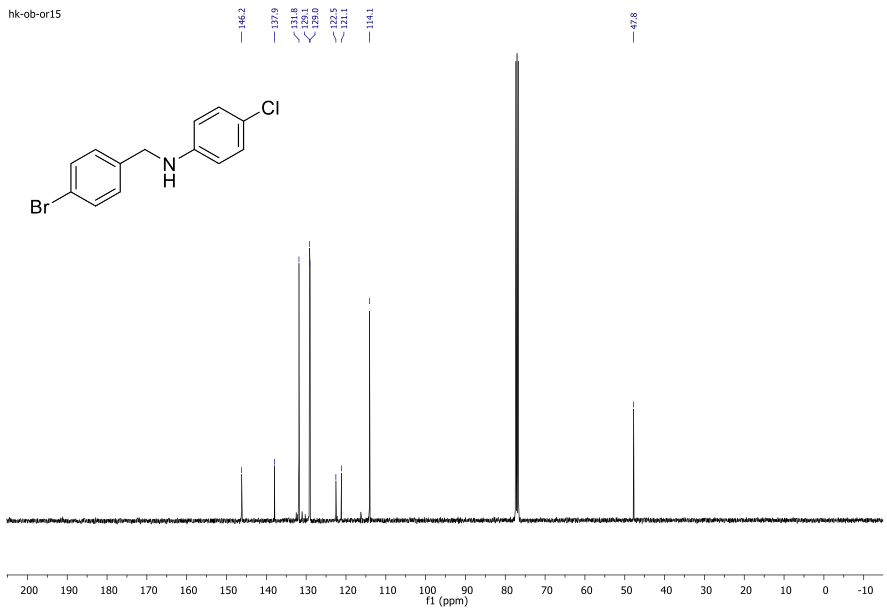

^{13}C NMR Spectrum of *N*-(4-bromobenzyl)-4-chloroaniline (**2a**) in $CDCl_3$

***N*-(4-Bromobenzyl)-4-methylaniline (2b)**

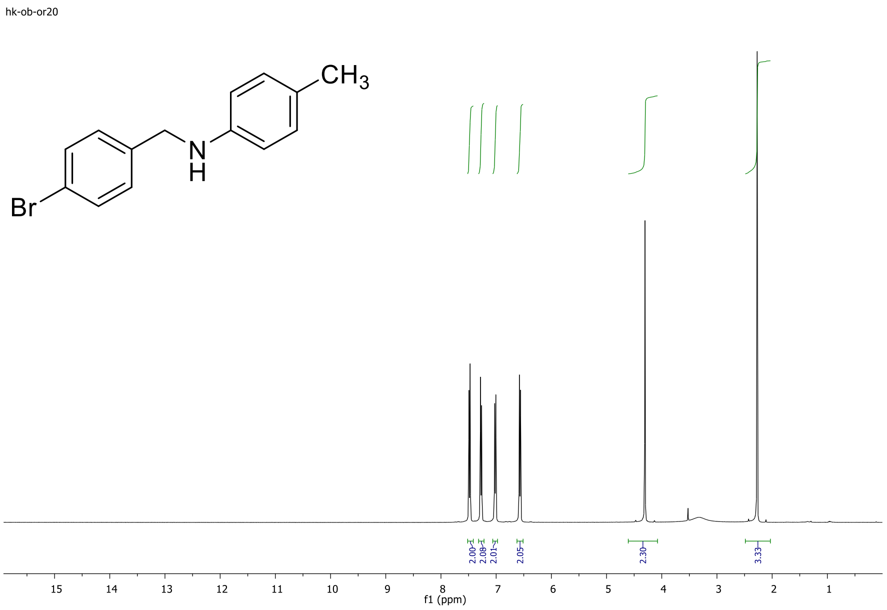

^{1}H NMR Spectrum of *N*-(4-bromobenzyl)-4-methylaniline (**2b**) in $CDCl_3$

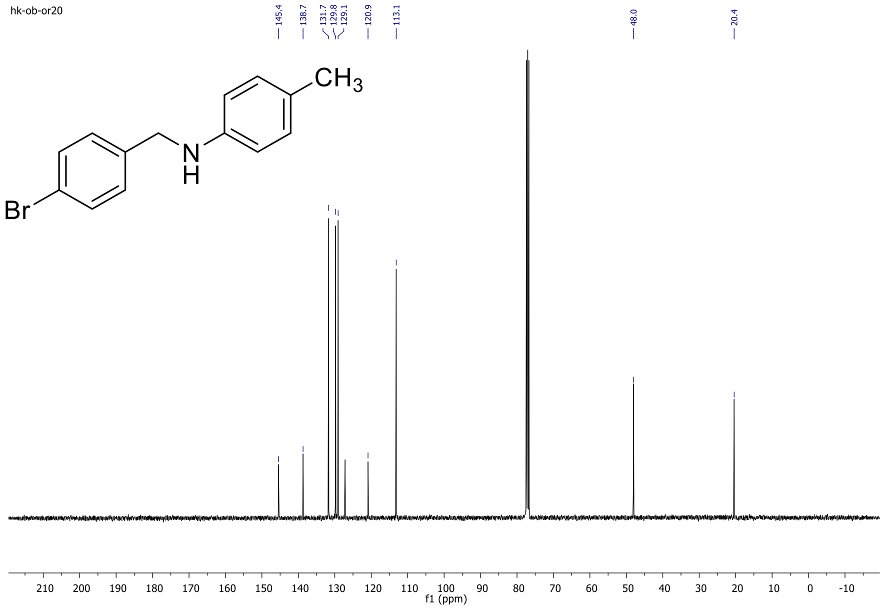

^{13}C NMR Spectrum of *N*-(4-bromobenzyl)-4-methylaniline (**2b**) in $CDCl_3$

4-Chloro-*N*-(4-chlorobenzyl)aniline (2c)

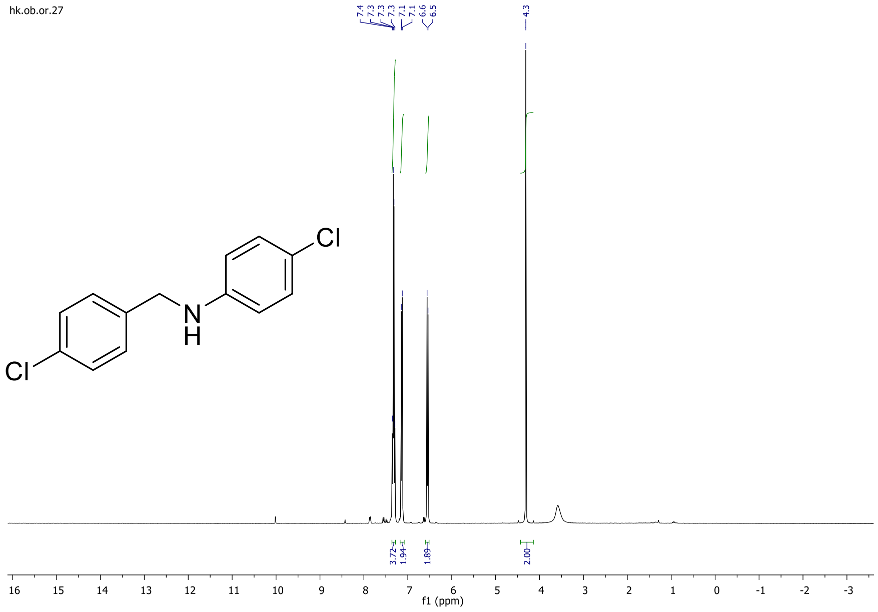

^{1}H NMR Spectrum of 4-chloro-*N*-(4-chlorobenzyl)aniline (**2c**) in $CDCl_3$

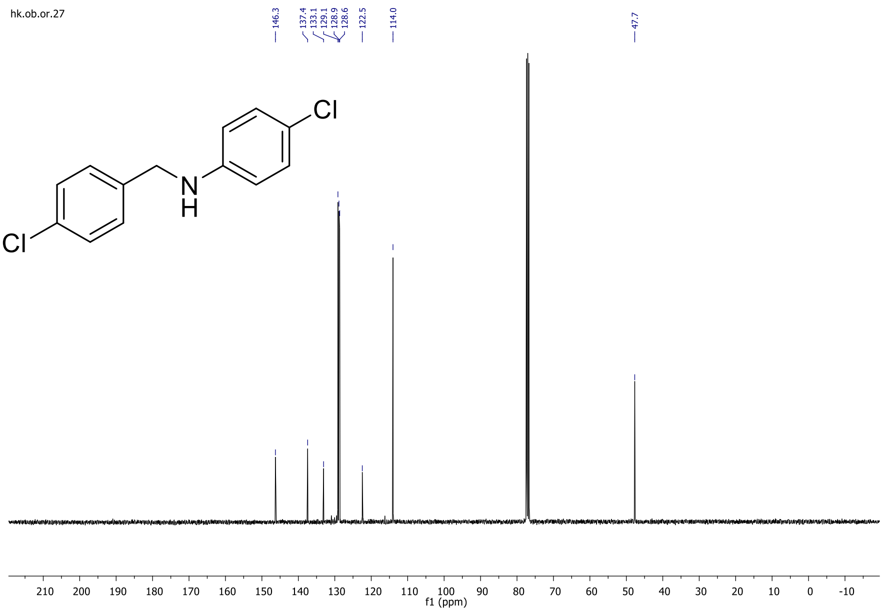

^{13}C NMR Spectrum of 4-chloro-*N*-(4-chlorobenzyl)aniline (**2c**) in $CDCl_3$

N-(4-Chlorobenzyl)-4-methylaniline (2d)

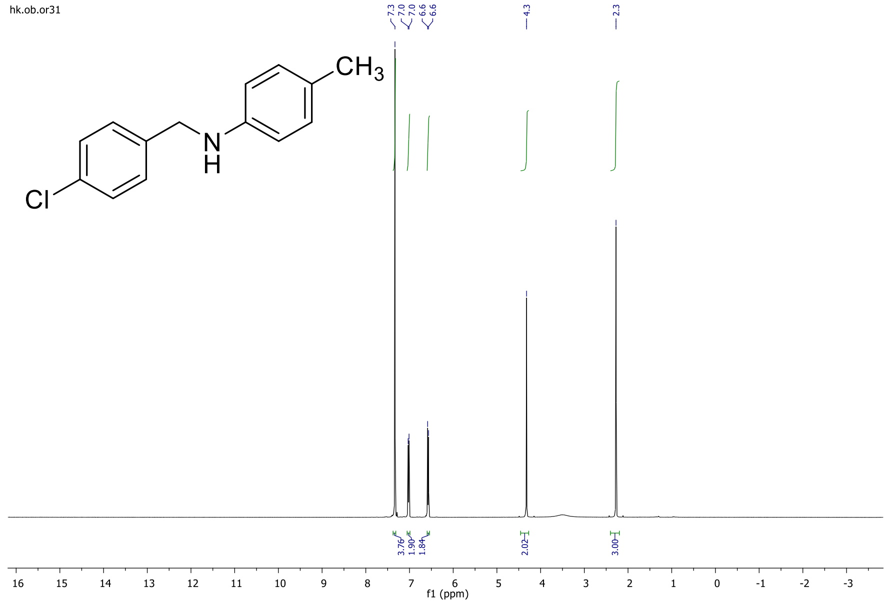

^{1}H NMR Spectrum of *N*-(4-chlorobenzyl)-4-methylaniline (**2d**) in $CDCl_3$

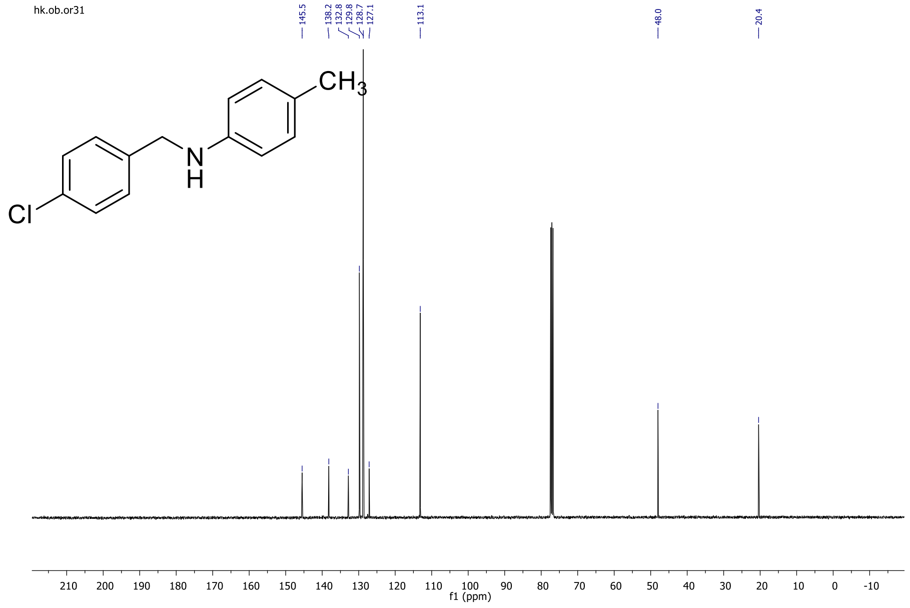

^{13}C NMR Spectrum of *N*-(4-chlorobenzyl)-4-methylaniline (**2d**) in $CDCl_3$

4-Chloro-*N*-(4-nitrobenzyl)aniline (2e)

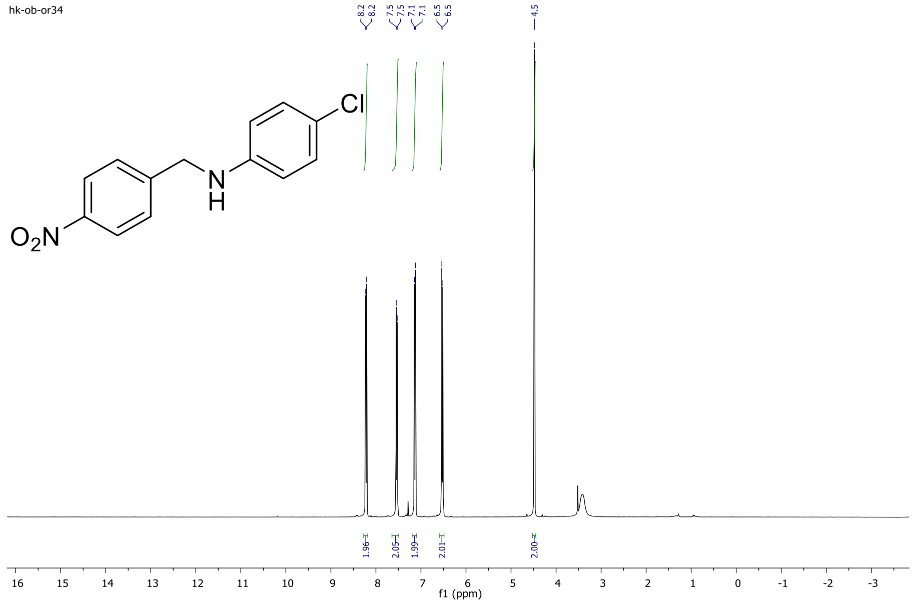

^{1}H NMR Spectrum of 4-chloro-*N*-(4-nitrobenzyl)aniline (**2e**) in $CDCl_3$

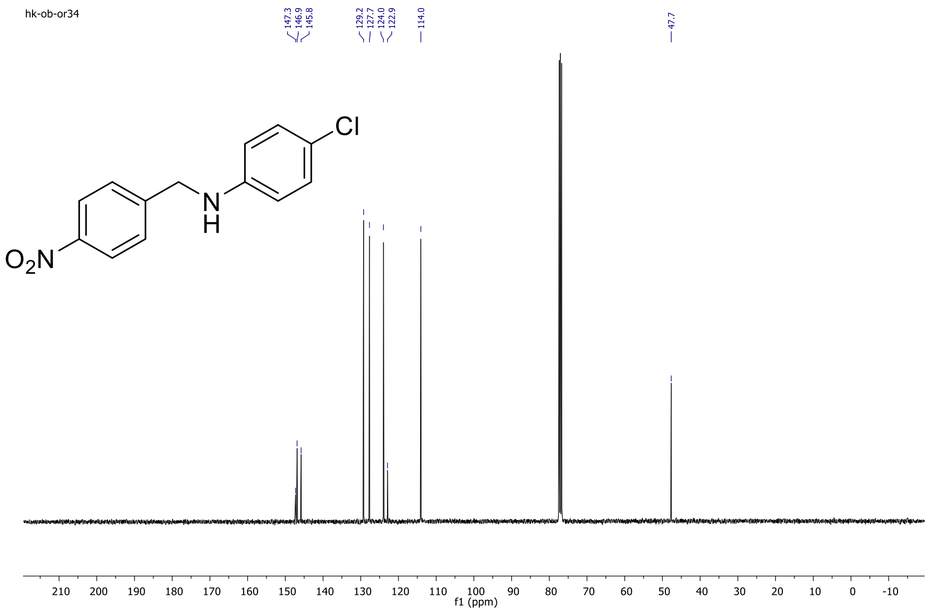

^{13}C NMR Spectrum of 4-chloro-*N*-(4-nitrobenzyl)aniline (**2e**) in $CDCl_3$

4-Methyl-*N*-(4-nitrobenzyl)aniline (2f)

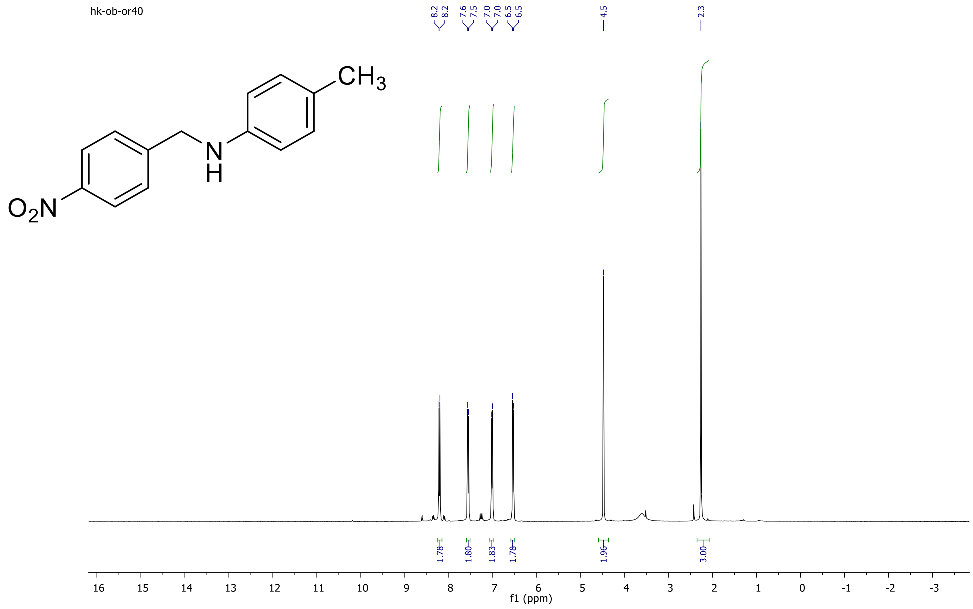

^{1}H NMR Spectrum of 4-methyl-*N*-(4-nitrobenzyl)aniline (**2f**) in $CDCl_3$

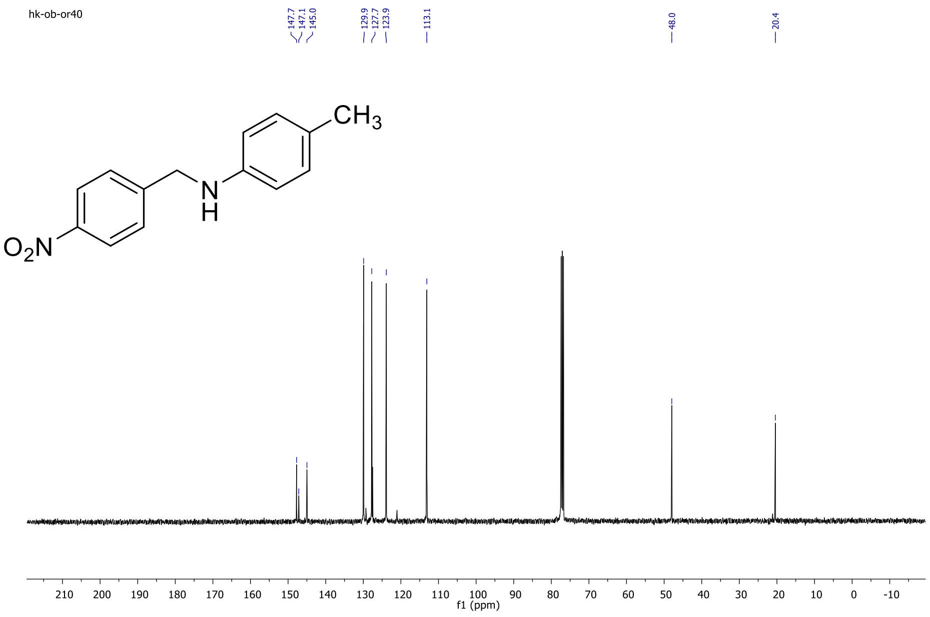

^{13}C NMR Spectrum of 4-methyl-*N*-(4-nitrobenzyl)aniline (**2f**) in $CDCl_3$

4-Chloro-*N*-(4-methoxybenzyl)aniline (2g)

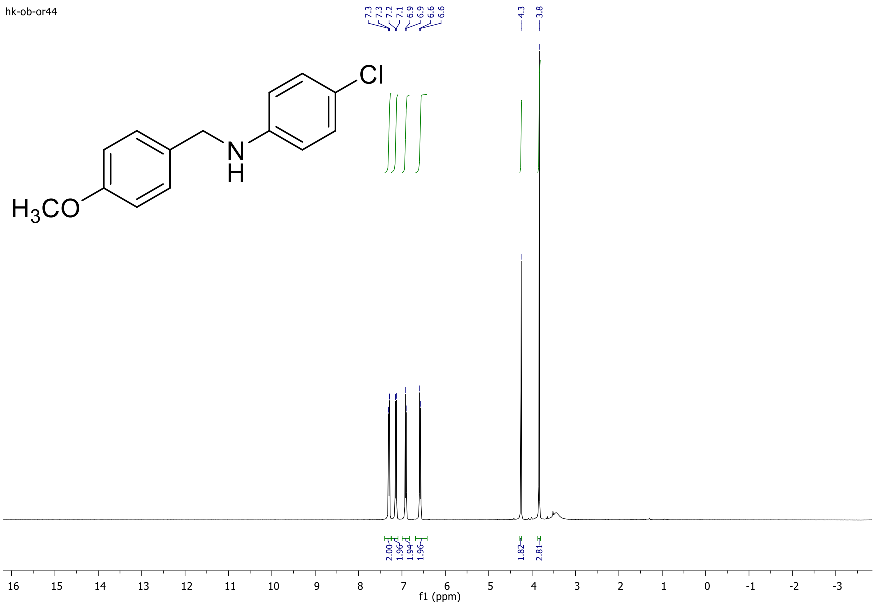

^{1}H NMR Spectrum of 4-chloro-*N*-(4-methoxybenzyl)aniline (**2g**) in $CDCl_3$

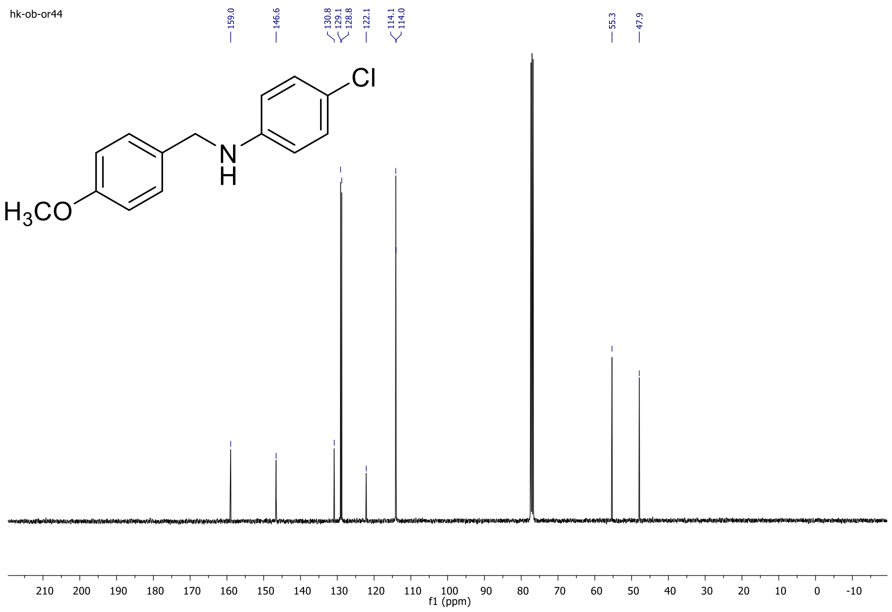

^{13}C NMR Spectrum of 4-chloro-*N*-(4-methoxybenzyl)aniline (**2g**) in $CDCl_3$

N-(4-Methoxybenzyl)-4-methylaniline (2h)

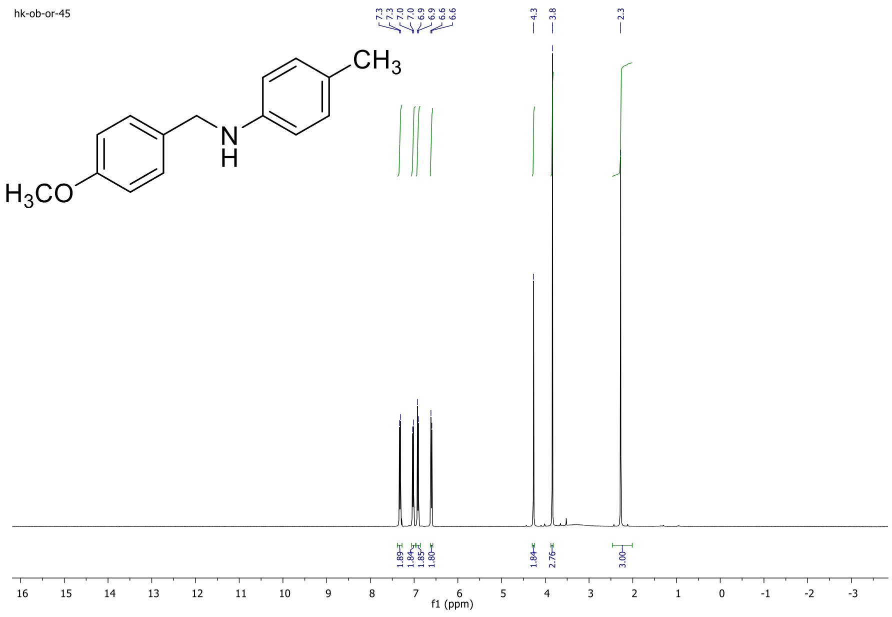

^{1}H NMR Spectrum of *N*-(4-methoxybenzyl)-4-methylaniline (**2h**) in $CDCl_3$

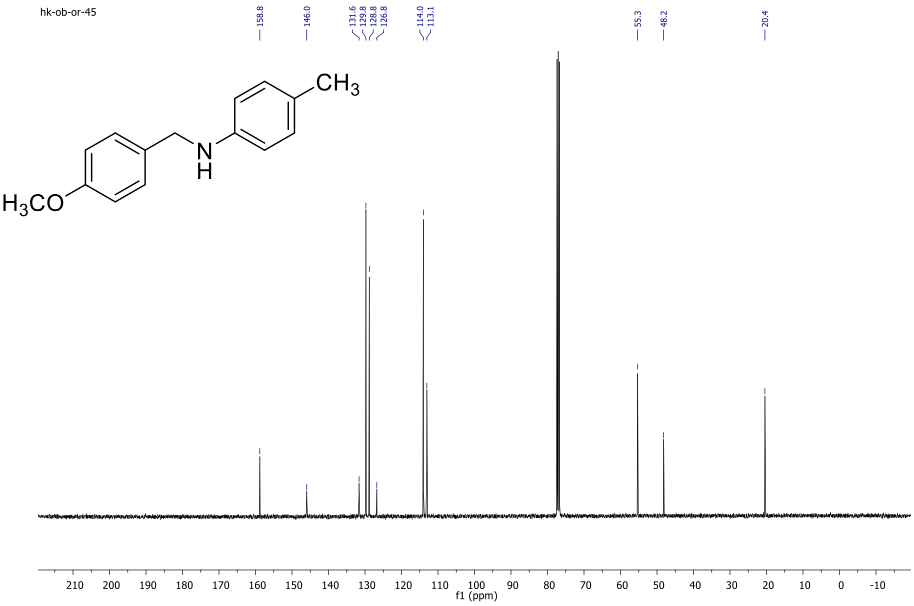

^{13}C NMR Spectrum of *N*-(4-methoxybenzyl)-4-methylaniline (**2h**) in $CDCl_3$

N-(4-Bromobenzyl)-2-(1H-indol-3-yl)ethan-1-amine (2i)

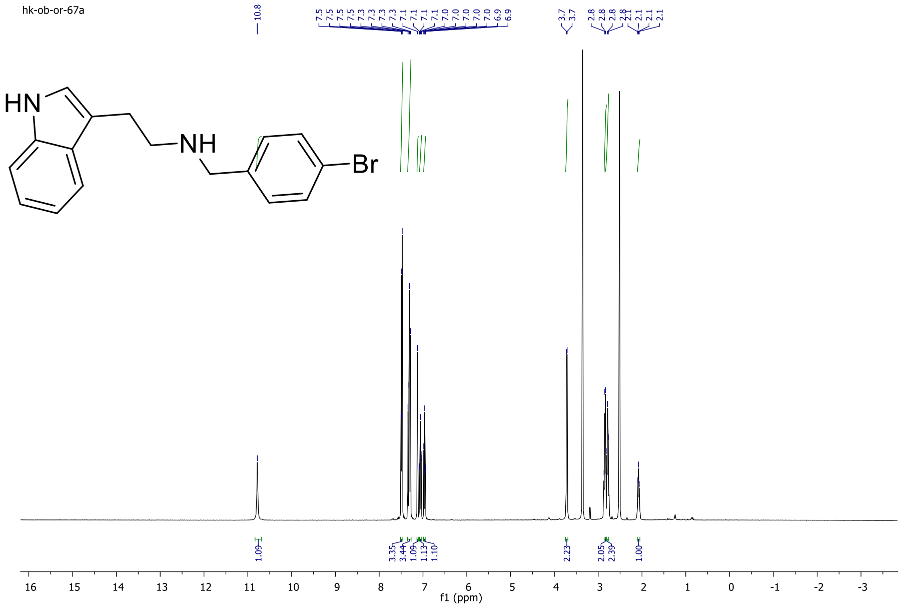

^{1}H NMR Spectrum of *N*-(4-bromobenzyl)-2-(1*H*-indol-3-yl)ethan-1-amine (**2i**) in DMSO-d_6

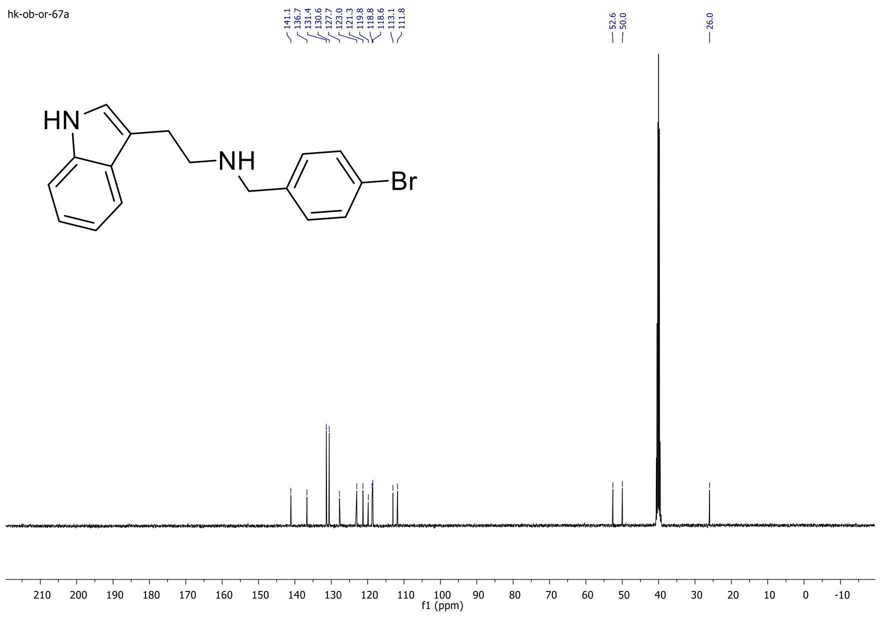

^{13}C NMR Spectrum of *N*-(4-bromobenzyl)-2-(1*H*-indol-3-yl)ethan-1-amine (**2i**) in DMSO-d_6

Data of sulfonamides 4a-i

N-(4-Bromobenzyl)-*N*-(4-chlorophenyl)-4-methylbenzenesulfonamide (4a)

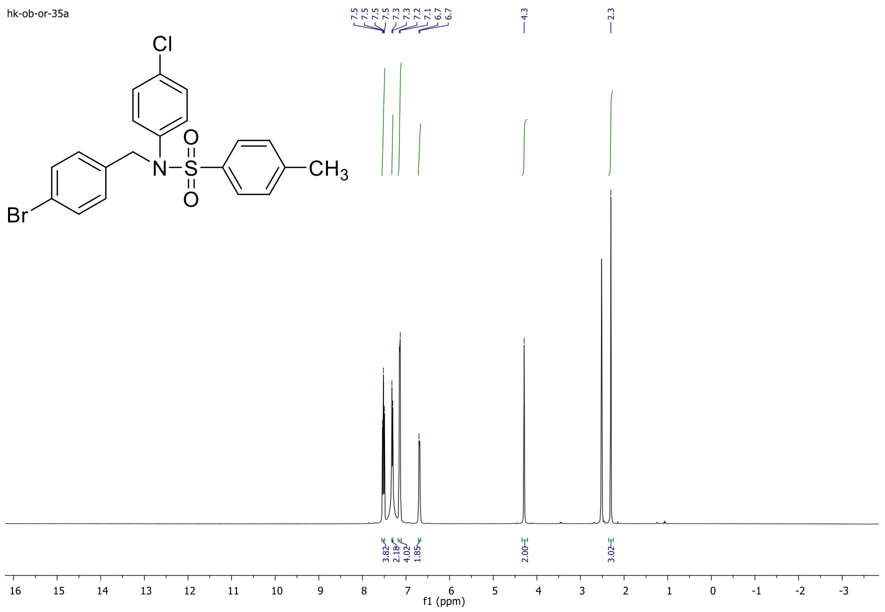

^{1}H NMR Spectrum of *N*-(4-bromobenzyl)-*N*-(4-chlorophenyl)-4-methylbenzenesulfonamide (**4a**) in DMSO-d_6

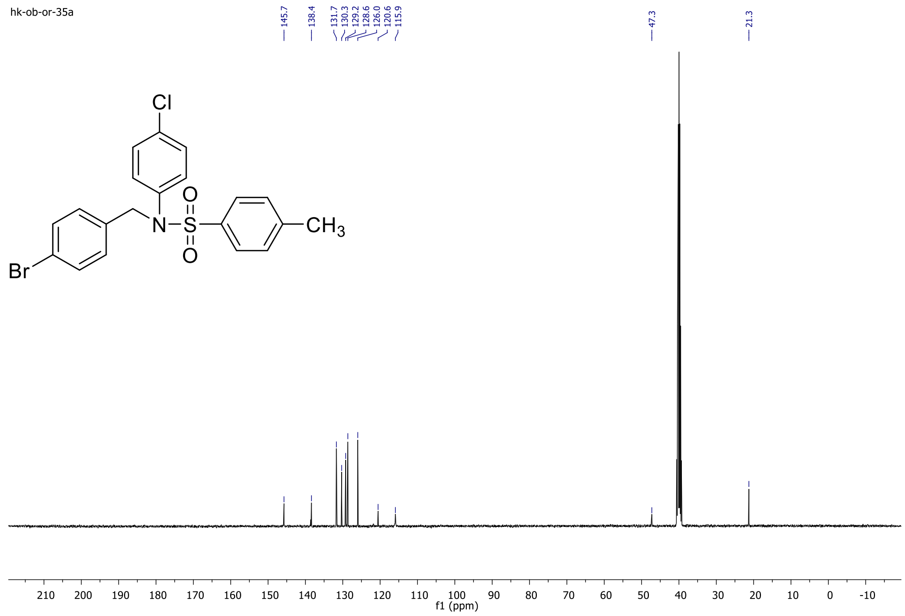

^{13}C NMR Spectrum of *N*-(4-bromobenzyl)-*N*-(4-chlorophenyl)-4-methylbenzenesulfonamide (**4a**) in DMSO-d_6

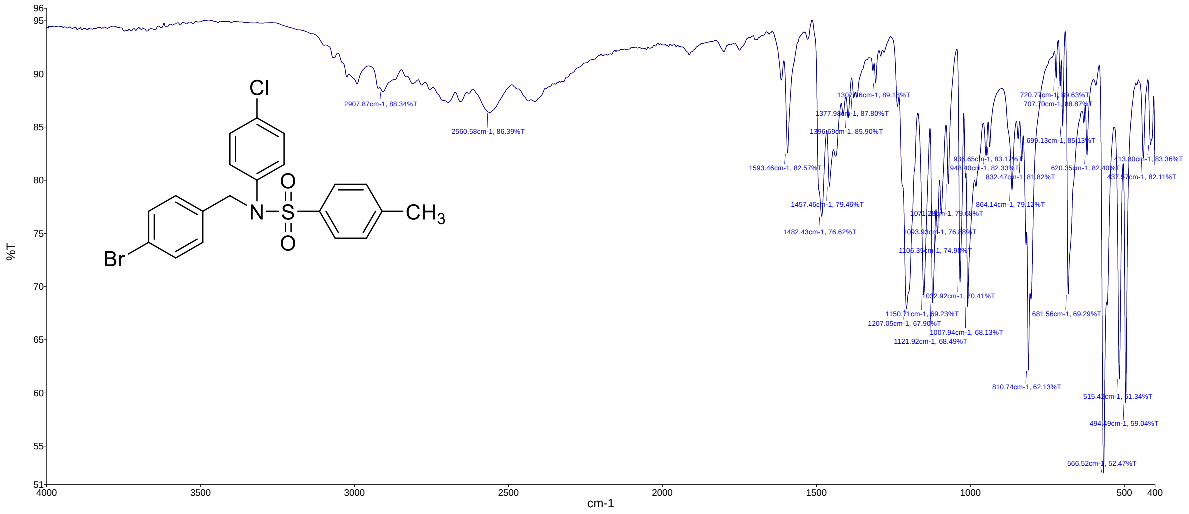

FTIR of *N*-(4-bromobenzyl)-*N*-(4-chlorophenyl)-4-methylbenzenesulfonamide (**4a**)

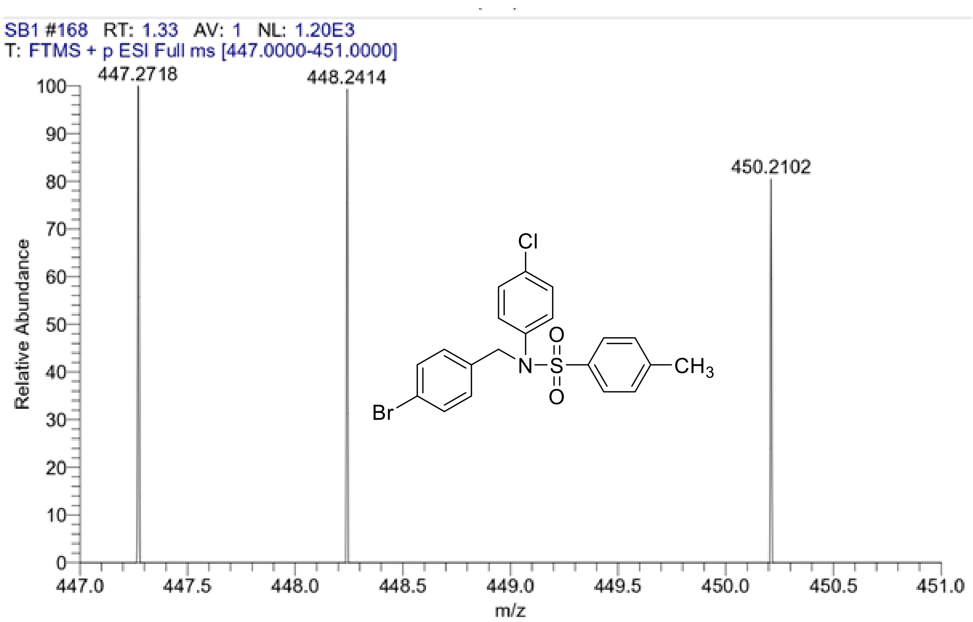

HRMS of *N*-(4-bromobenzyl)-*N*-(4-chlorophenyl)-4-methylbenzenesulfonamide (**4a**) **(**HRMS (ESI$^+$, FTMS) *m/z* for $C_{20}H_{17}BrClNO_2S$ $[M]^+$ calcd. 448. 9852, found 448.2414 $[M]^+$)

_N_-(4-Bromobenzyl)-4-methyl-_N_-(p-tolyl)benzenesulfonamide (4b)

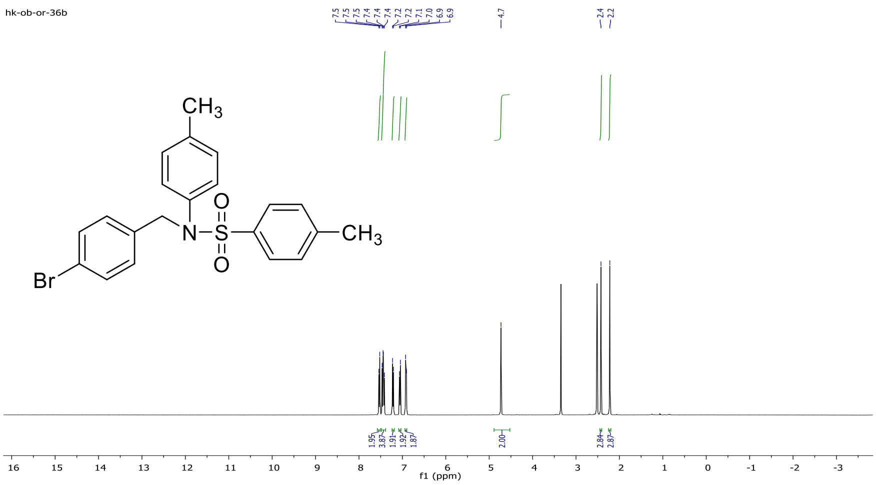

^{1}H NMR Spectrum of *N*-(4-bromobenzyl)-4-methyl-*N*-(p-tolyl)benzenesulfonamide (**4b**) in DMSO-d_6

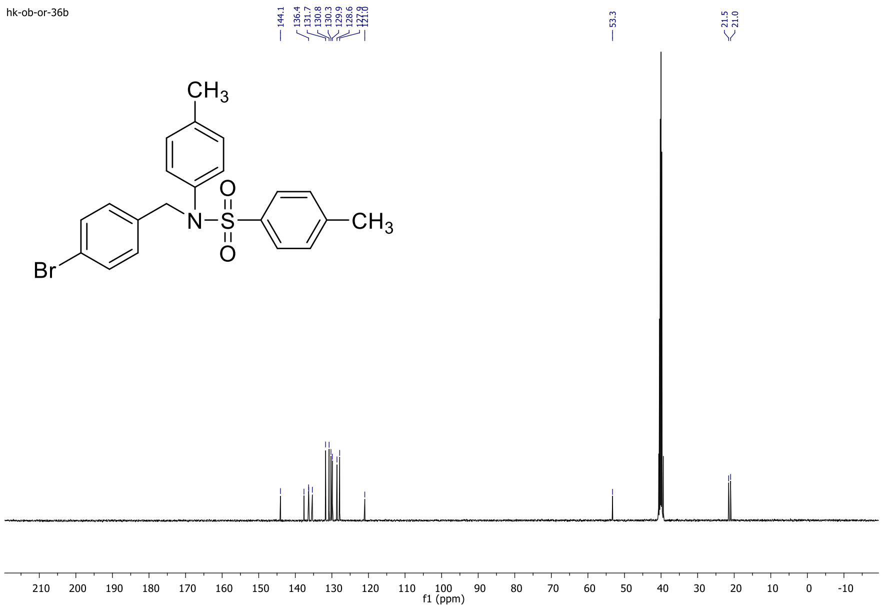

^{13}C NMR Spectrum of *N*-(4-bromobenzyl)-4-methyl-*N*-(p-tolyl)benzenesulfonamide (**4b**) in DMSO-d_6

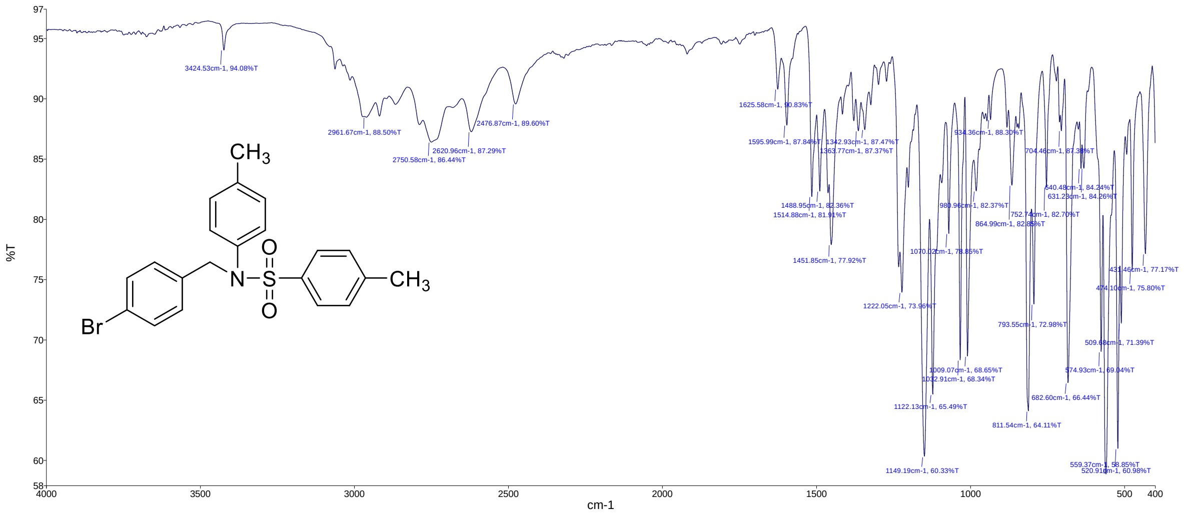

FTIR of *N*-(4-bromobenzyl)-4-methyl-*N*-(p-tolyl)benzenesulfonamide (**4b**)

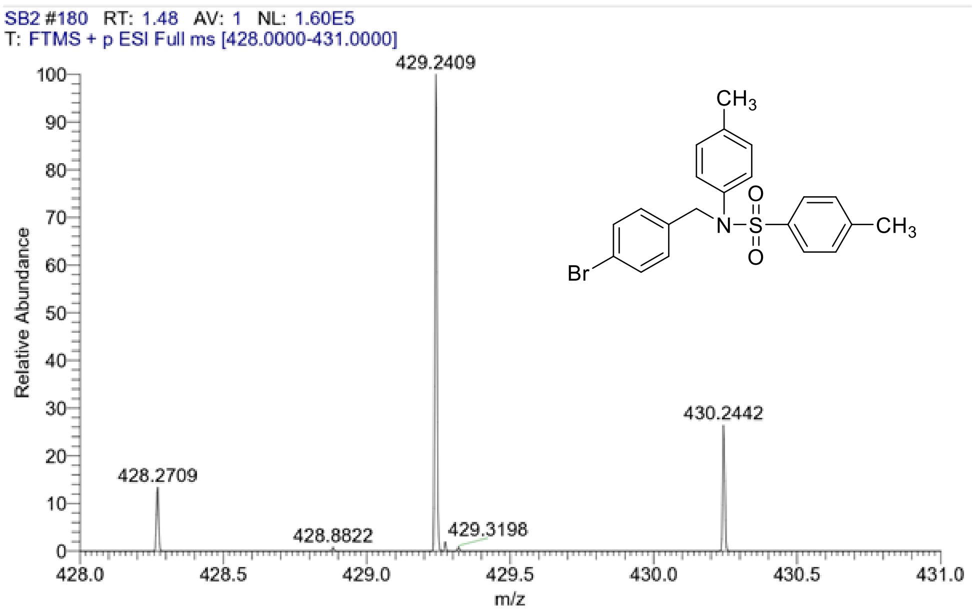

HRMS of *N*-(4-bromobenzyl)-4-methyl-*N*-(p-tolyl)benzenesulfonamide (**4b**) (HRMS (ESI$^+$, FTMS) *m/z* for $C_{21}H_{20}BrNO_2S$ $[M]^+$ calcd. 429.0398, found 429.2409 $[M]^+$)

_N_-(4-Chlorobenzyl)-_N_-(4-chlorophenyl)-4-methylbenzenesulfonamide (4c)

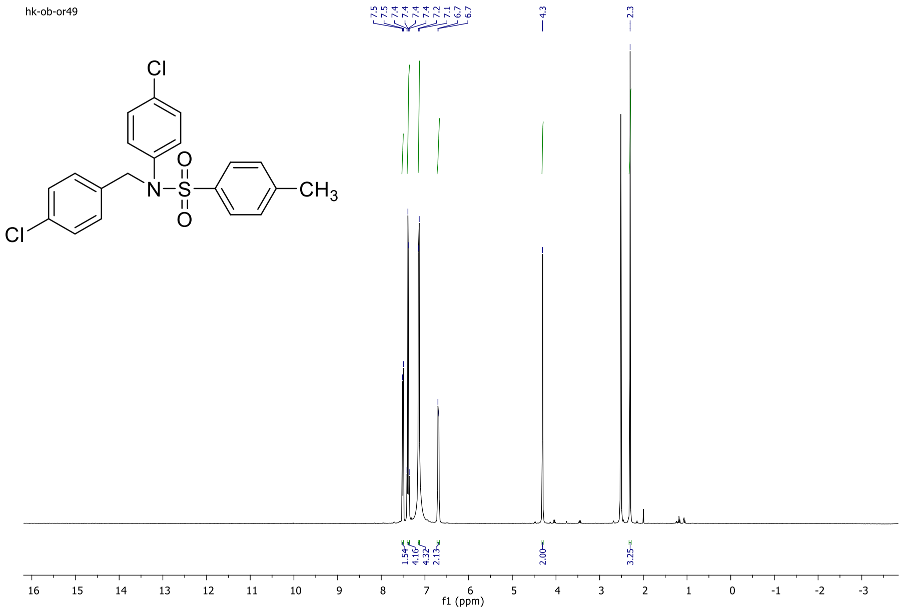

^{1}H NMR Spectrum of *N*-(4-chlorobenzyl)-*N*-(4-chlorophenyl)-4-methylbenzenesulfonamide (**4c**) in DMSO-d_6

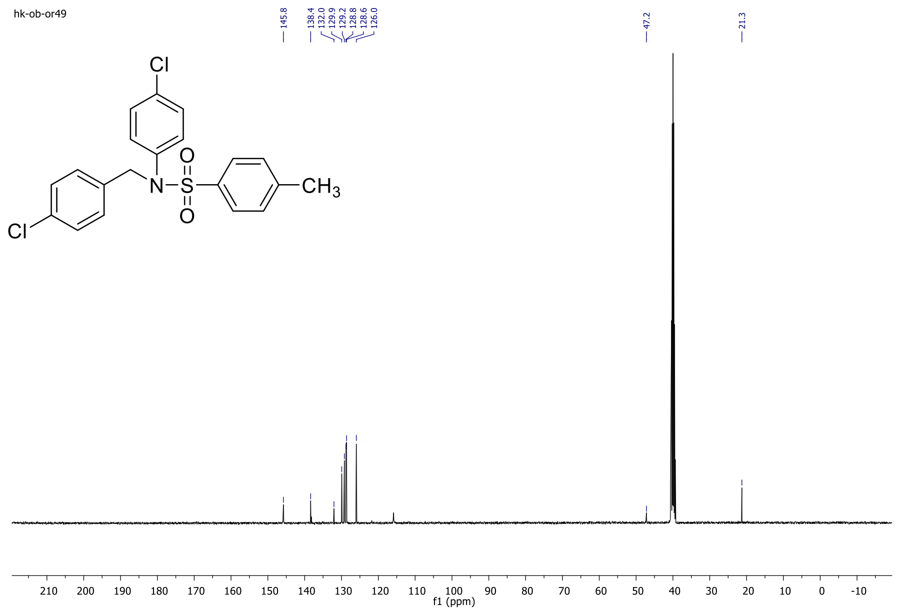

^{13}C NMR Spectrum of *N*-(4-chlorobenzyl)-*N*-(4-chlorophenyl)-4-methylbenzenesulfonamide (**4c**) in DMSO-d_6

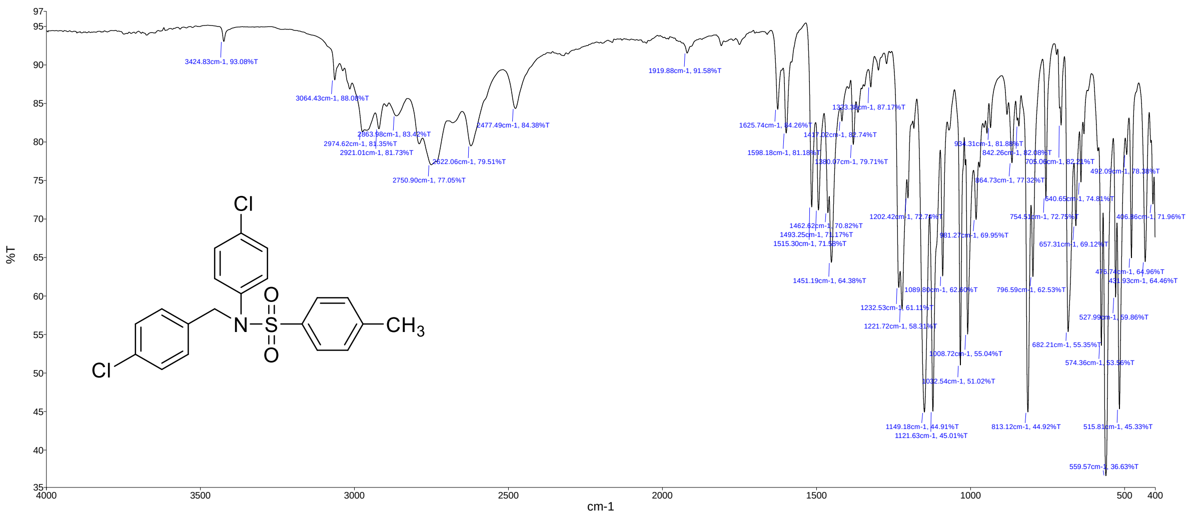

FTIR of of *N*-(4-chlorobenzyl)-*N*-(4-chlorophenyl)-4-methylbenzenesulfonamide (**4c**)

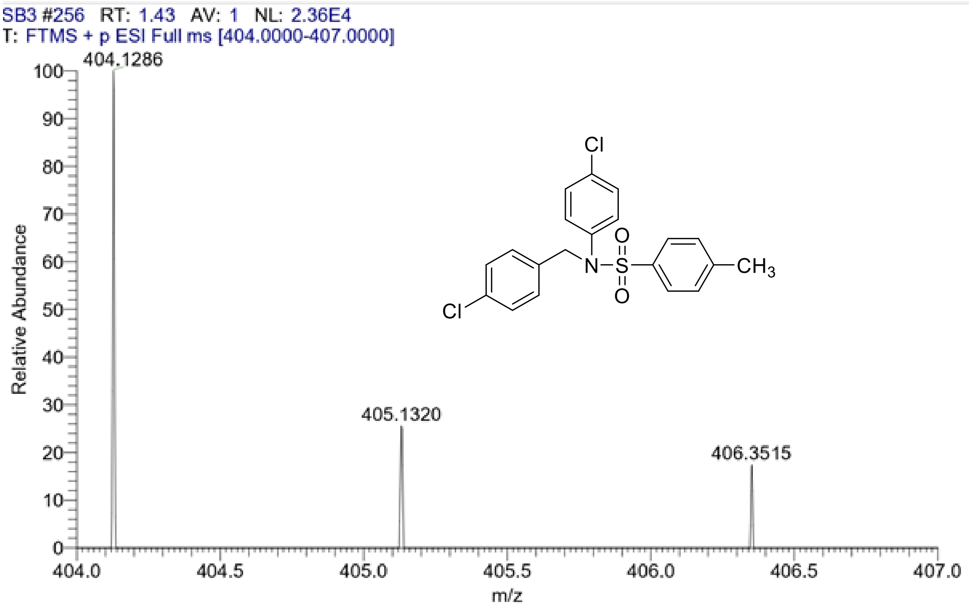

HRMS of of *N*-(4-chlorobenzyl)-*N*-(4-chlorophenyl)-4-methylbenzenesulfonamide (**4c**) (HRMS (ESI$^+$, FTMS) m/z**:** $[M]^+$ calcd for $C_{20}H_{17}Cl_2NO_2S$: 405,0357; found: 405.1320 $[M]^+$).

N-(4-Chlorobenzyl)-4-methyl-*N*-(*p*-tolyl)benzenesulfonamide (4d)

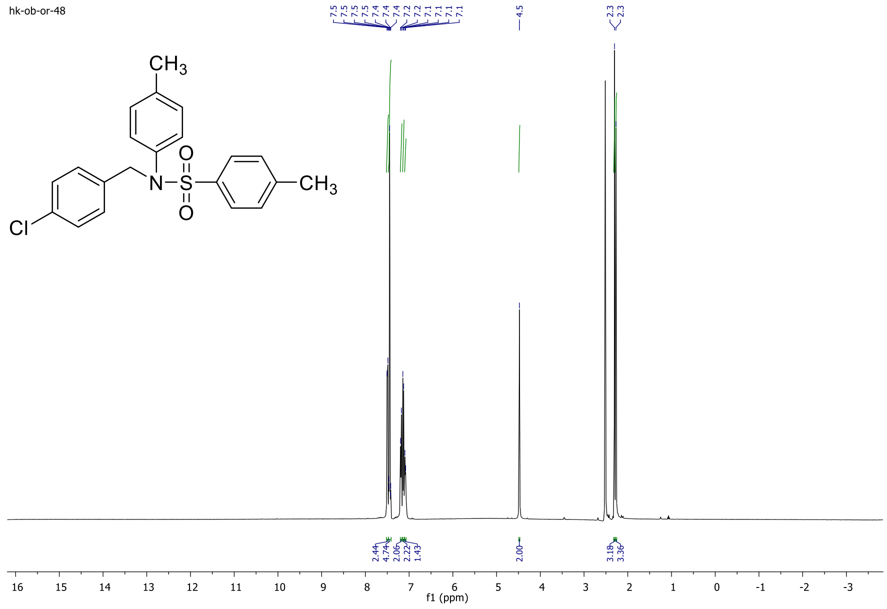

^{1}H NMR Spectrum of *N*-(4-chlorobenzyl)-4-methyl-*N*-(*p*-tolyl)benzenesulfonamide (**4d**) in DMSO-d_6

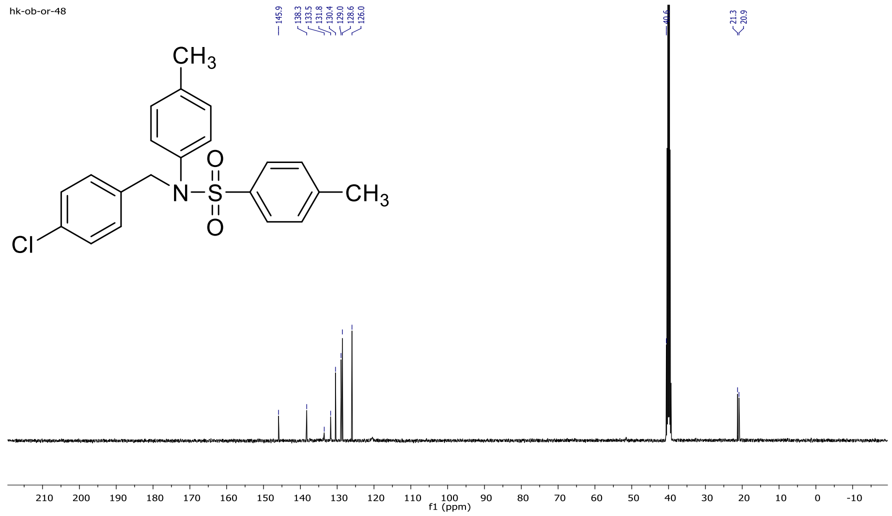

^{13}C NMR Spectrum of *N*-(4-chlorobenzyl)-4-methyl-*N*-(*p*-tolyl)benzenesulfonamide (**4d**) in DMSO-d_6

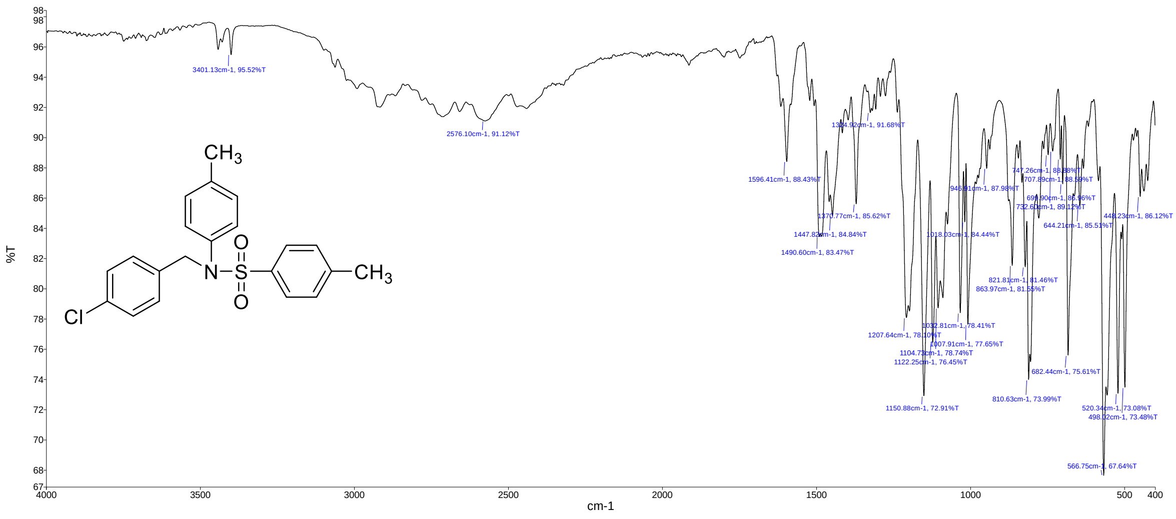

FTIR of *N*-(4-chlorobenzyl)-4-methyl-*N*-(*p*-tolyl)benzenesulfonamide (**4d**)

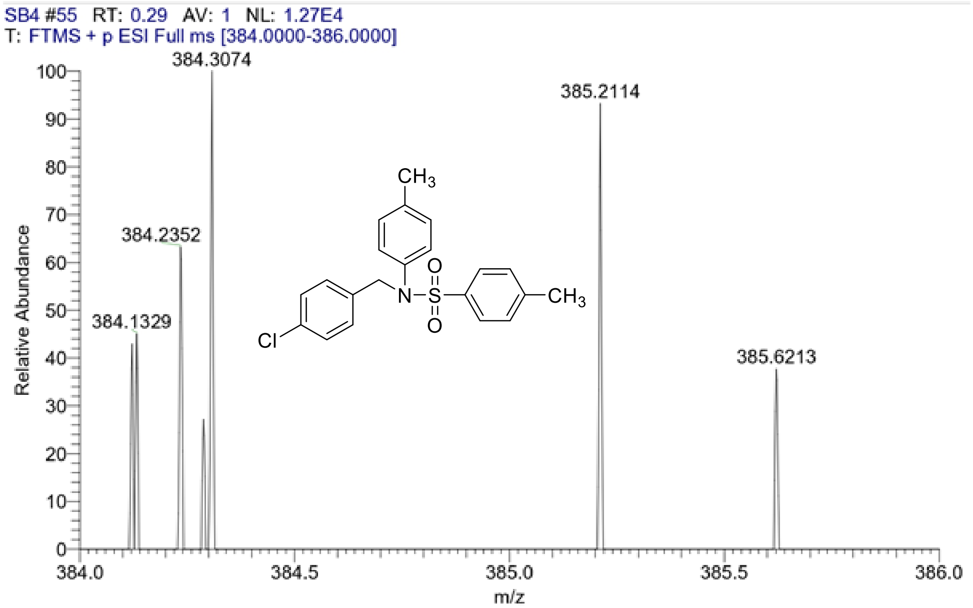

HRMS of *N*-(4-chlorobenzyl)-4-methyl-*N*-(*p*-tolyl)benzenesulfonamide (**4d**) (HRMS (ESI$^+$, FTMS) m/z: $[M]^+$ calcd for $C_{21}H_{20}ClNO_2S$: 385,0903; found: 385.2114 $[M]^+$).

_N_-(4-Chlorophenyl)-4-methyl-_N_-(4-nitrobenzyl)benzenesulfonamide (4e)

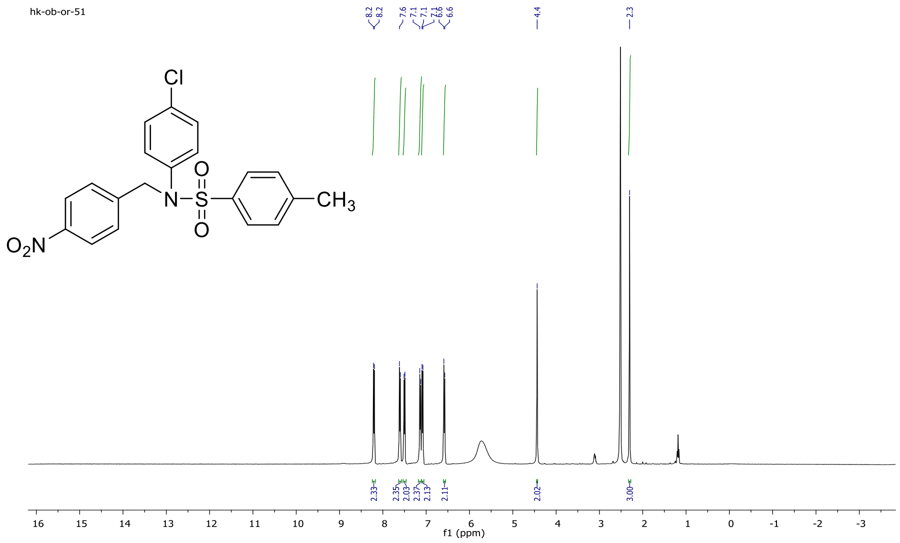

^{1}H NMR Spectrum of *N*-(4-chlorophenyl)-4-methyl-*N*-(4-nitrobenzyl)benzenesulfonamide (**4e**) in DMSO-d_6

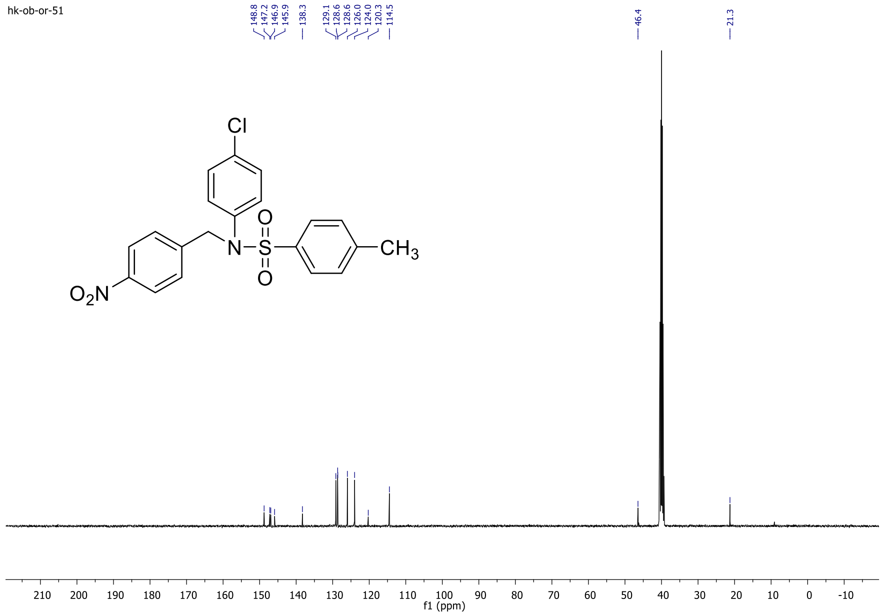

^{13}C NMR Spectrum of *N*-(4-chlorophenyl)-4-methyl-*N*-(4-nitrobenzyl)benzenesulfonamide (**4e**) in DMSO-d_6

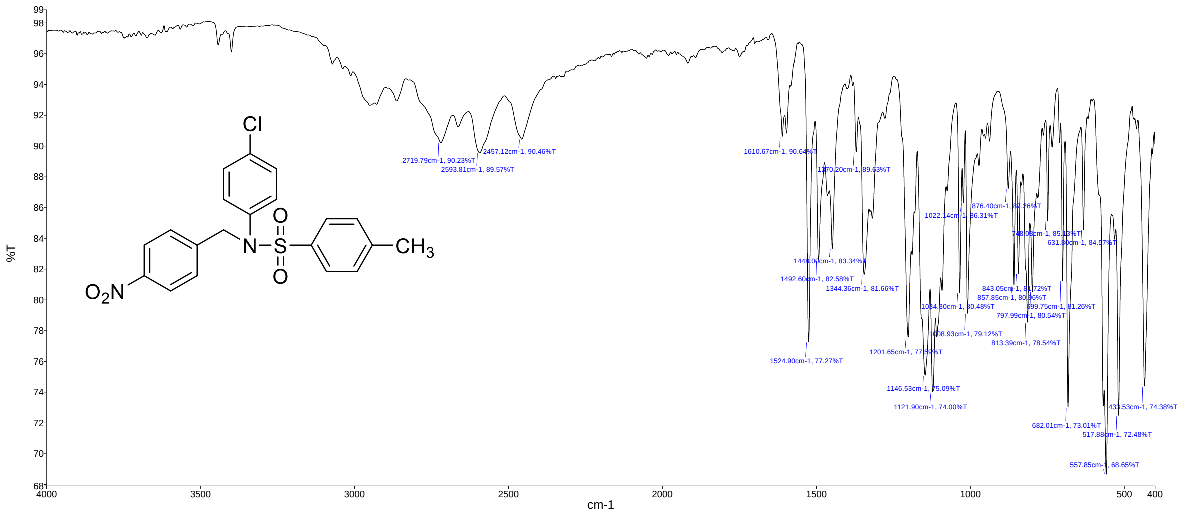

FTIR of *N*-(4-chlorophenyl)-4-methyl-*N*-(4-nitrobenzyl)benzenesulfonamide (**4e**)

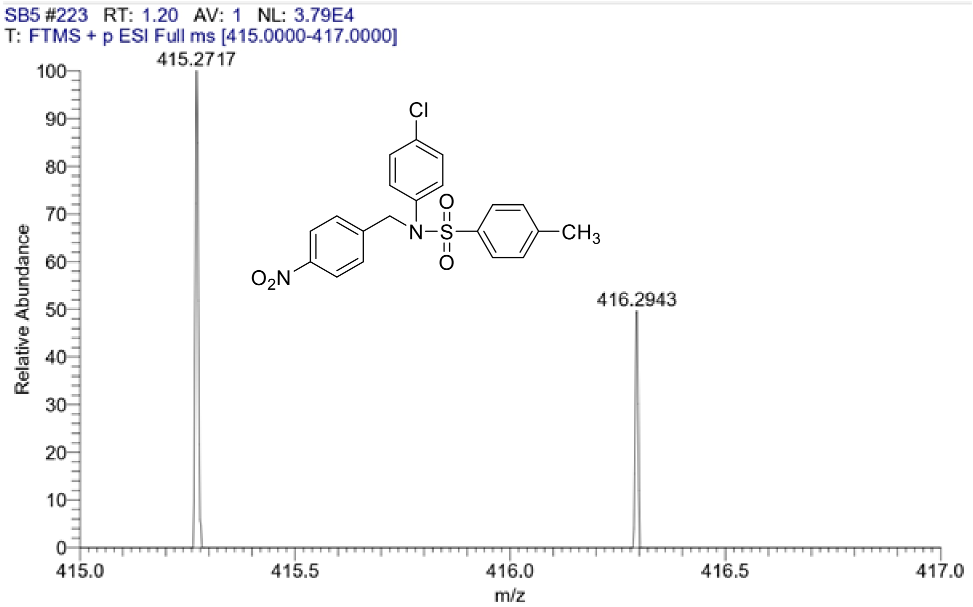

HRMS of *N*-(4-chlorophenyl)-4-methyl-*N*-(4-nitrobenzyl)benzenesulfonamide (**4e**) (HRMS (ESI$^+$, FTMS) m/z: [M]$^+$ calcd for $C_{20}H_{17}ClN_2O_4S$: 416,0598; found: 416.2943 [M]$^+$).

4-Methyl-*N*-(4-nitrobenzyl)-*N*-(*p*-tolyl)benzenesulfonamide (4f)

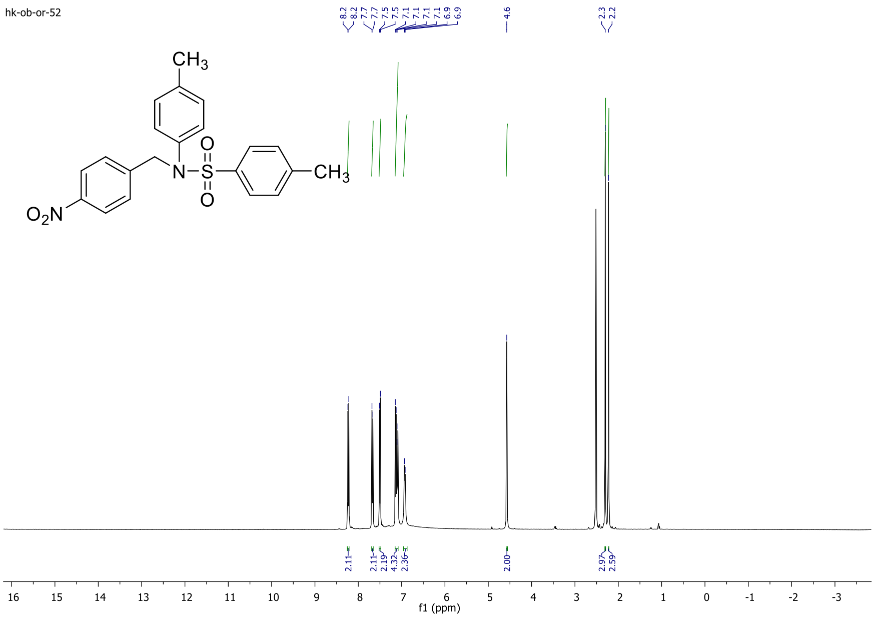

^{1}H NMR Spectrum of 4-methyl-*N*-(4-nitrobenzyl)-*N*-(*p*-tolyl)benzenesulfonamide (**4f**) in DMSO-d_6

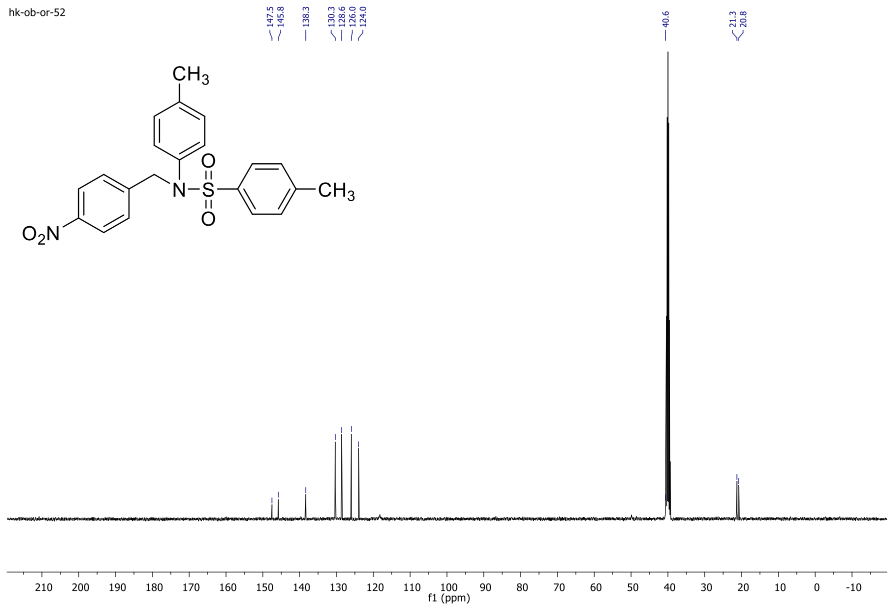

^{13}C NMR Spectrum of 4-methyl-*N*-(4-nitrobenzyl)-*N*-(*p*-tolyl)benzenesulfonamide (**4f)** in DMSO-d$_6$

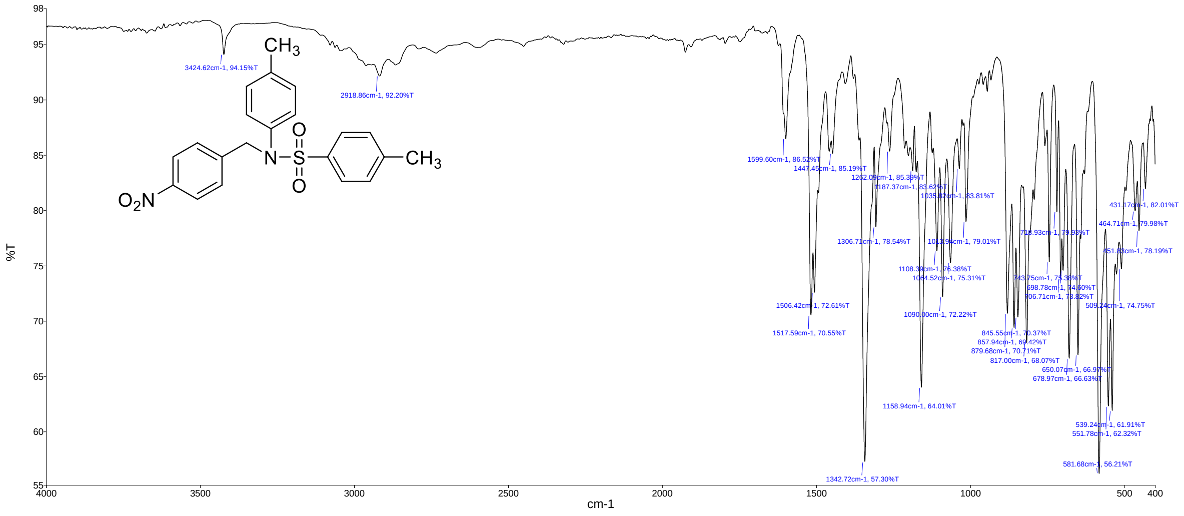

FTIR of 4-methyl-*N*-(4-nitrobenzyl)-*N*-(*p*-tolyl)benzenesulfonamide (**4f**)

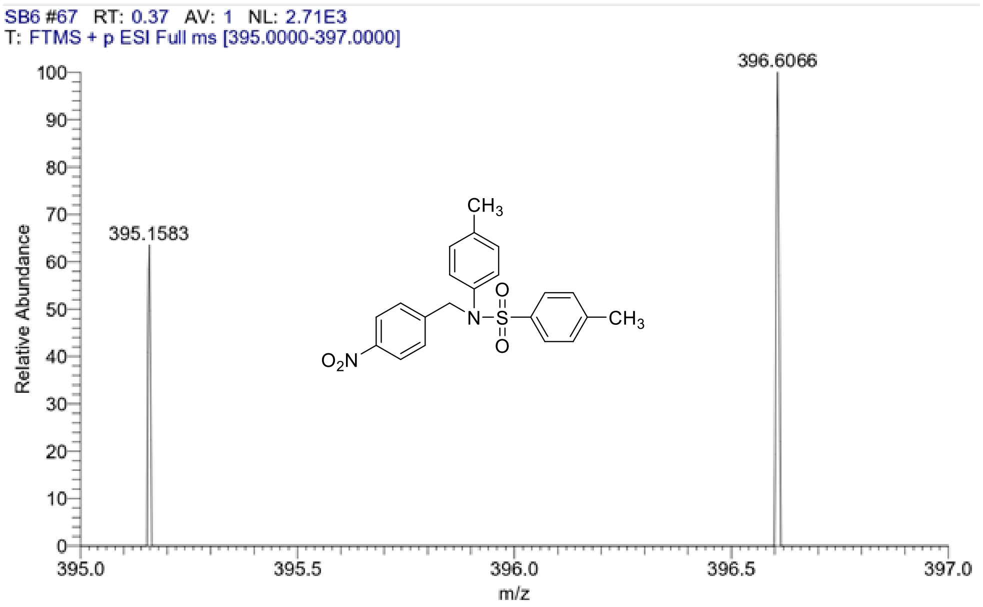

HRMS 4-methyl-*N*-(4-nitrobenzyl)-*N*-(*p*-tolyl)benzenesulfonamide (**4f**) (HRMS (ESI$^+$, FTMS) m/z: $[M]^+$ calcd for $C_{21}H_{20}N_2O_4S$: 396,1144; found: 396.6066 $[M]^+$).

_N_-(4-Chlorophenyl)-_N_-(4-methoxybenzyl)-4-methylbenzenesulfonamide (4g)

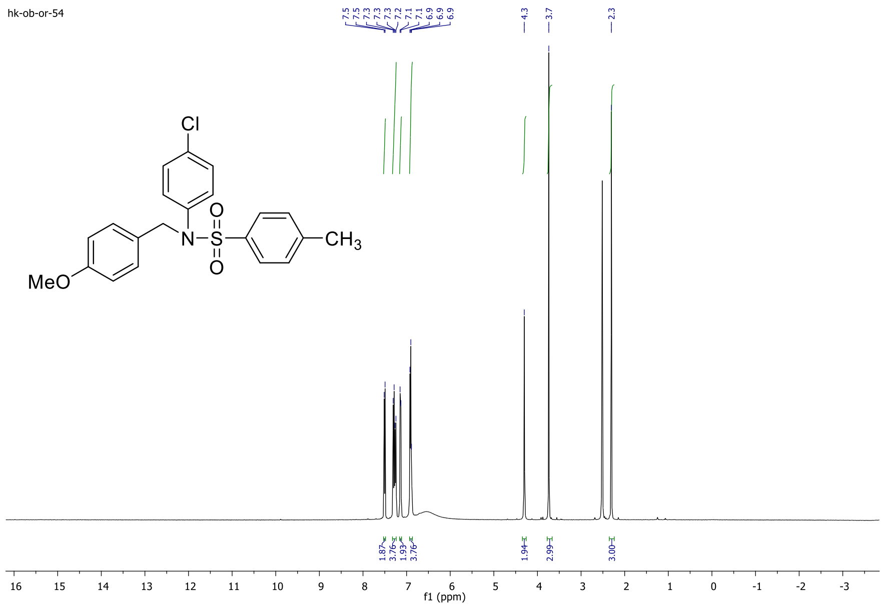

^{1}H NMR Spectrum of *N*-(4-chlorophenyl)-*N*-(4-methoxybenzyl)-4-methylbenzenesulfonamide (**4g**) in DMSO-d_6

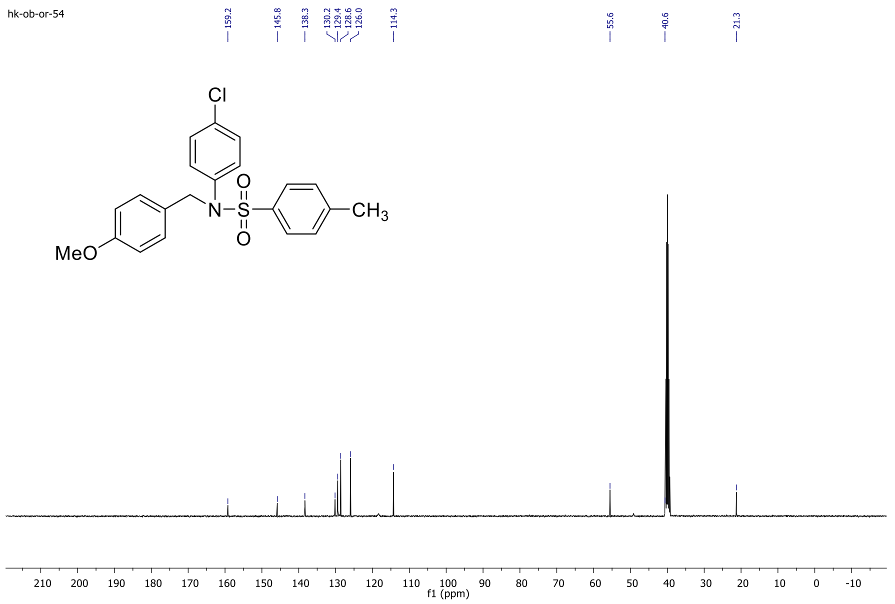

^{13}C NMR Spectrum of *N*-(4-chlorophenyl)-*N*-(4-methoxybenzyl)-4-methylbenzenesulfonamide (**4g**) in DMSO-d_6

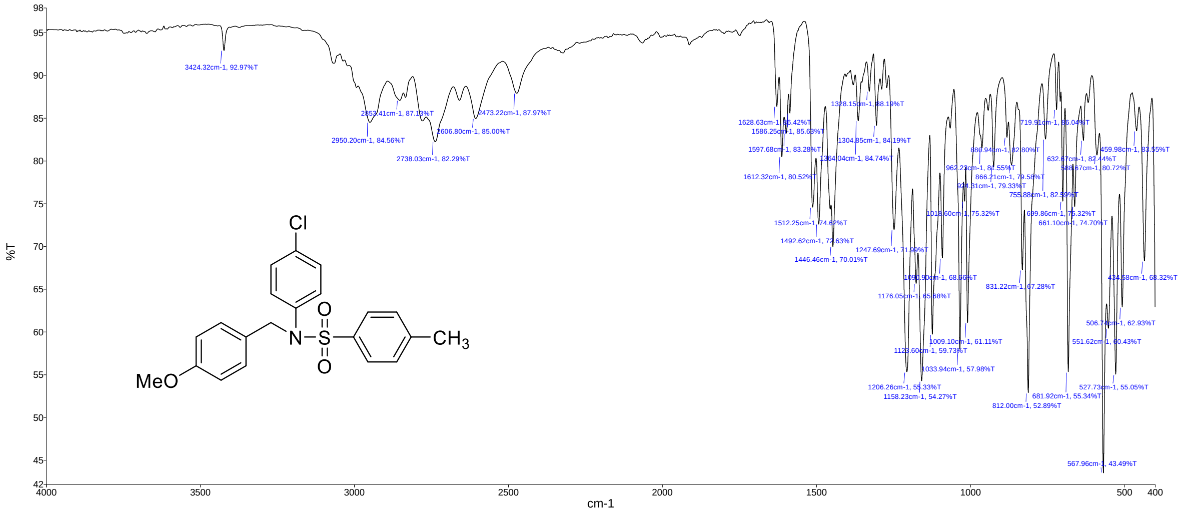

FTIR of *N*-(4-chlorophenyl)-*N*-(4-methoxybenzyl)-4-methylbenzenesulfonamide (**4g**)

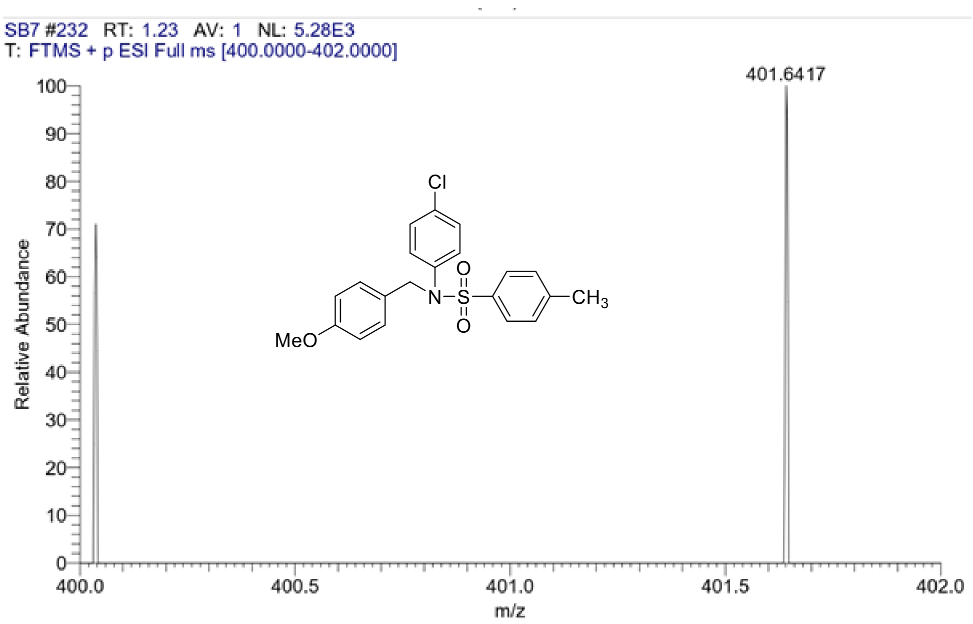

HRMS of of *N*-(4-chlorophenyl)-*N*-(4-methoxybenzyl)-4-methylbenzenesulfonamide (**4g**) (HRMS (ESI+, FTMS) m/z: [M]+ calcd for $C_{21}H_{20}ClNO_3S$: 401,0852; found: 401.6417 [M]+).

***N*-(4-Methoxybenzyl)-4-methyl-*N*-(*p*-tolyl)benzenesulfonamide (4h)**

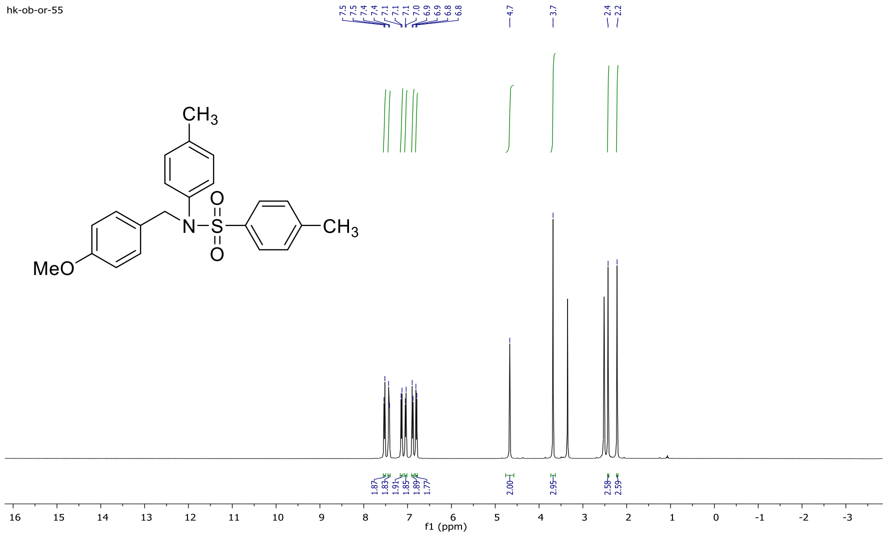

^{1}H NMR Spectrum of *N*-(4-methoxybenzyl)-4-methyl-*N*-(*p*-tolyl)benzenesulfonamide (**4h**) in DMSO-d_6

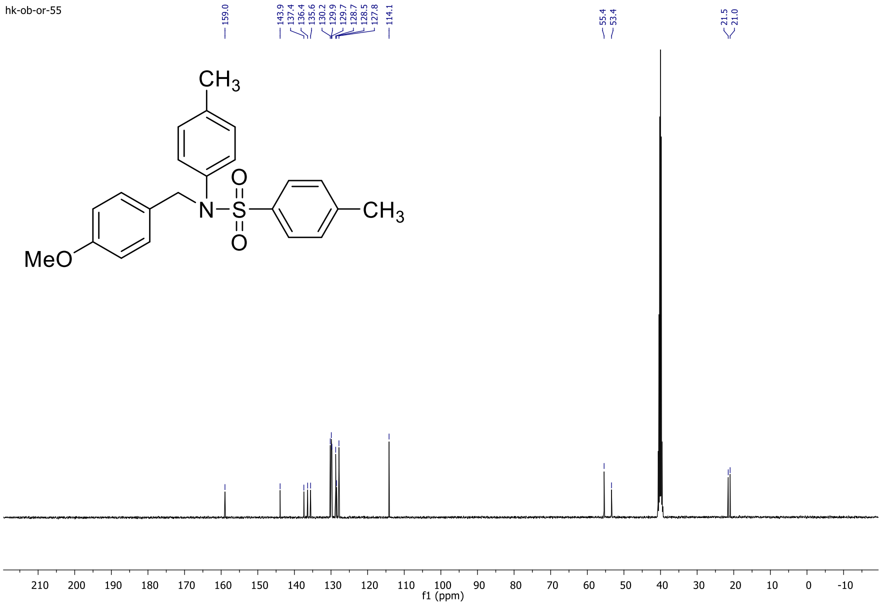

^{13}C NMR Spectrum of *N*-(4-methoxybenzyl)-4-methyl-*N*-(*p*-tolyl)benzenesulfonamide (**4h**) in DMSO-d$_6$

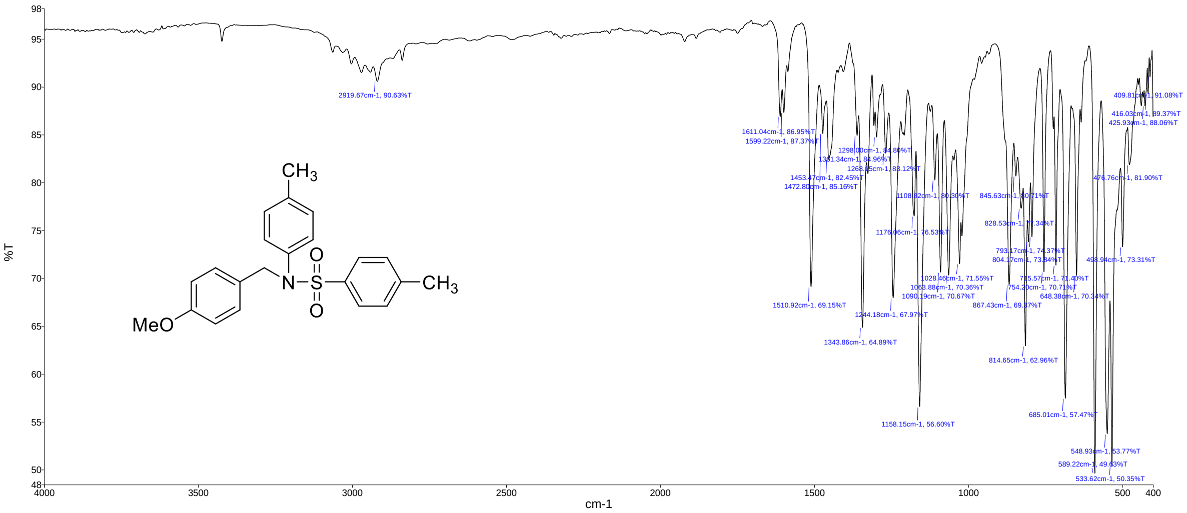

FTIR of of *N*-(4-methoxybenzyl)-4-methyl-*N*-(*p*-tolyl)benzenesulfonamide (**4h**)

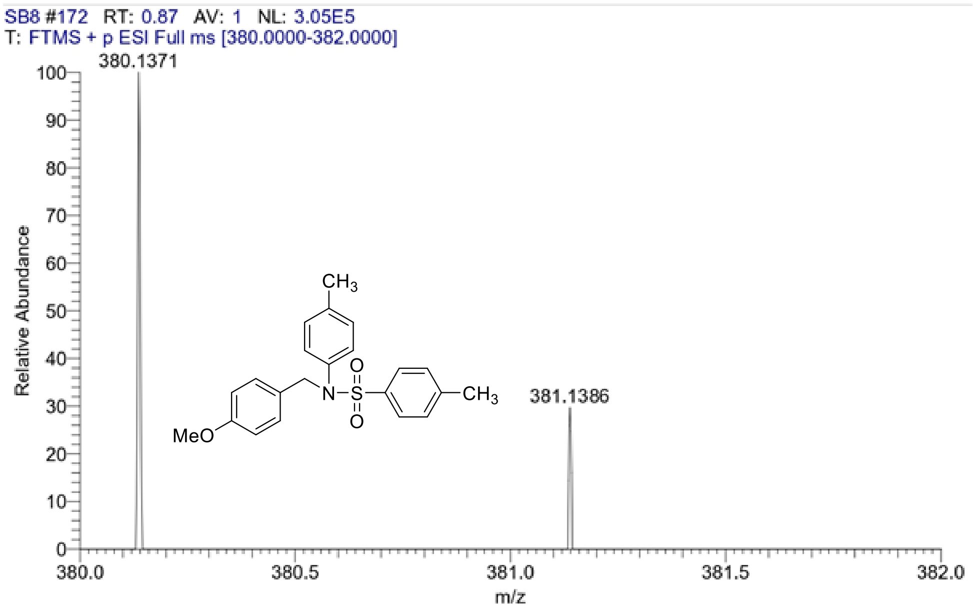

HRMS of *N*-(4-methoxybenzyl)-4-methyl-*N*-(*p*-tolyl)benzenesulfonamide (**4h**) (HRMS (ESI$^+$, FTMS) m/z:[M]$^+$ calcd for $C_{22}H_{23}NO_3S$:381.1399; found: 381.1386 [M]$^+$).

N-(2-(1*H*-indol-3-yl)ethyl)-N-(4-bromobenzyl)-4-methylbenzenesulfonamide (4i)

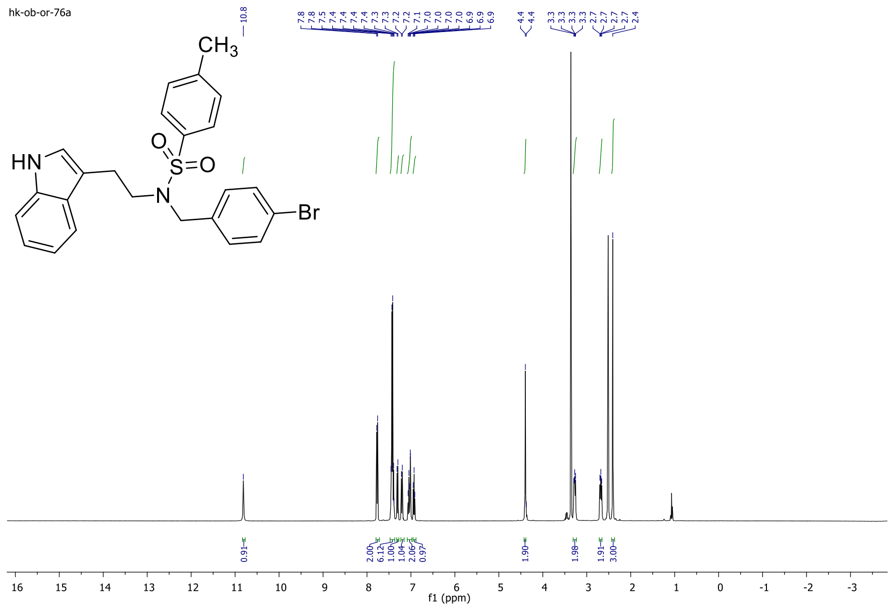

^{1}H NMR Spectrum of N-(2-(1*H*-indol-3-yl)ethyl)-N-(4-bromobenzyl)-4-methylbenzenesulfonamide (**4i**) in DMSO-d_6

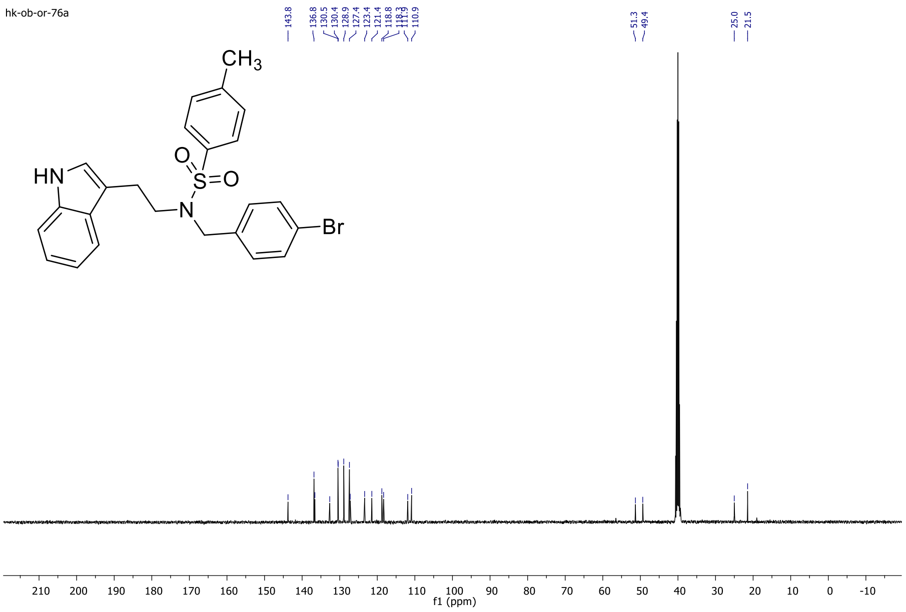

^{13}C NMR Spectrum of N-(2-(1*H*-indol-3-yl)ethyl)-N-(4-bromobenzyl)-4-methylbenzenesulfonamide (**4i**) in DMSO-d_6

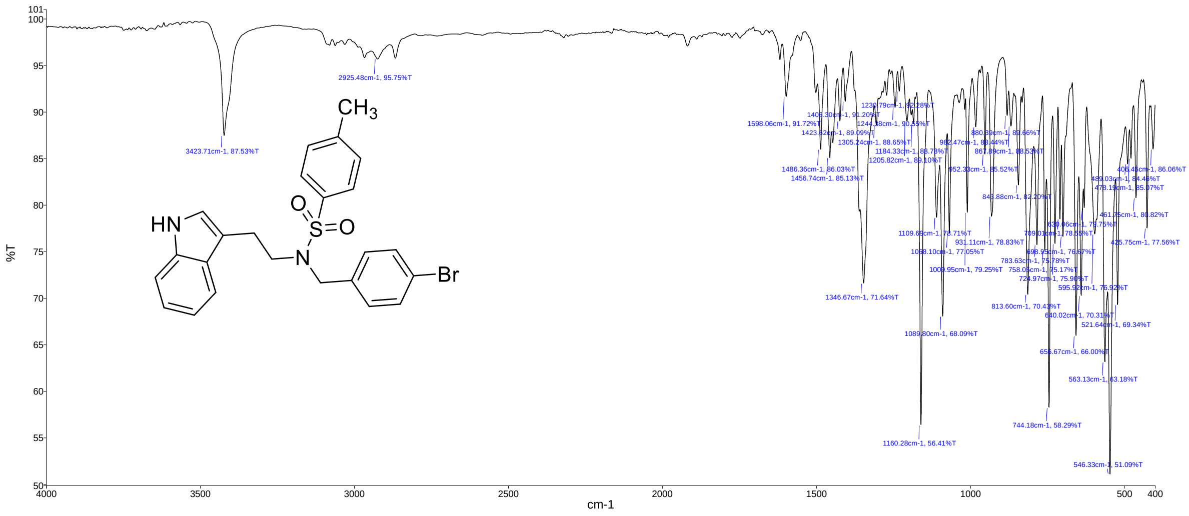

FTIR of *N*-(2-(1*H*-indol-3-yl)ethyl)-*N*-(4-bromobenzyl)-4-methylbenzenesulfonamide (**4i**)

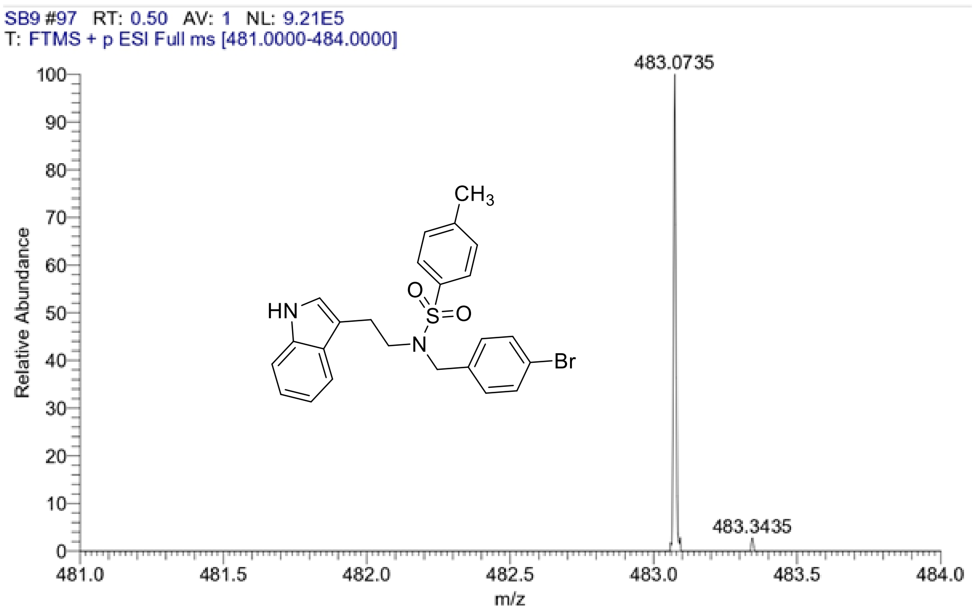

HRMS of N-(2-(1*H*-indol-3-yl)ethyl)-N-(4-bromobenzyl)-4-methylbenzenesulfonamide (**4i**) (HRMS (ESI$^+$, FTMS) m/z: $[M]^+$ calcd for $C_{24}H_{23}BrN_2O_2S$: 482,0664; found $[M+1]^+$**:** 483.0735 $[M]^+$).
